# Inhibitor of the Tyrosine Phosphatase STEP Reverses Cognitive Deficits in a Mouse Model of Alzheimer's Disease

**DOI:** 10.1371/journal.pbio.1001923

**Published:** 2014-08-05

**Authors:** Jian Xu, Manavi Chatterjee, Tyler D. Baguley, Jonathan Brouillette, Pradeep Kurup, Debolina Ghosh, Jean Kanyo, Yang Zhang, Kathleen Seyb, Chimezie Ononenyi, Ethan Foscue, George M. Anderson, Jodi Gresack, Gregory D. Cuny, Marcie A. Glicksman, Paul Greengard, TuKiet T. Lam, Lutz Tautz, Angus C. Nairn, Jonathan A. Ellman, Paul J. Lombroso

**Affiliations:** 1 Child Study Center, Yale University, New Haven, Connecticut, United States of America; 2 Department of Chemistry, Yale University, New Haven, Connecticut, United States of America; 3 Department of Molecular Biophysics and Biochemistry, Yale University, New Haven, Connecticut, United States of America; 4 Laboratory for Drug Discovery in Neurodegeneration and Department of Neurology, Brigham and Women's Hospital, Cambridge, Massachusetts, United States of America; 5 Department of Laboratory Medicine, Yale University, New Haven, Connecticut, United States of America; 6 Laboratory of Molecular and Cellular Neuroscience, The Rockefeller University, New York, New York, United States of America; 7 Infectious and Inflammatory Disease Center, Sanford-Burnham Medical Research Institute, La Jolla, California, United States of America; 8 Department of Psychiatry, Yale University, New Haven, Connecticut, United States of America; 9 Department of Neurobiology, Yale University, New Haven, Connecticut, United States of America; Stanford University, United States of America

## Abstract

STEP (STriatal-Enriched protein tyrosine Phosphatase) is a neuron-specific phosphatase that regulates N-methyl-D-aspartate receptor (NMDAR) and α-amino-3-hydroxy-5-methyl-4-isoxazolepropionic acid receptor (AMPAR) trafficking, as well as ERK1/2, p38, Fyn, and Pyk2 activity. STEP is overactive in several neuropsychiatric and neurodegenerative disorders, including Alzheimer's disease (AD). The increase in STEP activity likely disrupts synaptic function and contributes to the cognitive deficits in AD. AD mice lacking STEP have restored levels of glutamate receptors on synaptosomal membranes and improved cognitive function, results that suggest STEP as a novel therapeutic target for AD. Here we describe the first large-scale effort to identify and characterize small-molecule STEP inhibitors. We identified the benzopentathiepin 8-(trifluoromethyl)-1,2,3,4,5-benzopentathiepin-6-amine hydrochloride (known as TC-2153) as an inhibitor of STEP with an IC_50_ of 24.6 nM. TC-2153 represents a novel class of PTP inhibitors based upon a cyclic polysulfide pharmacophore that forms a reversible covalent bond with the catalytic cysteine in STEP. In cell-based secondary assays, TC-2153 increased tyrosine phosphorylation of STEP substrates ERK1/2, Pyk2, and GluN2B, and exhibited no toxicity in cortical cultures. Validation and specificity experiments performed in wild-type (WT) and STEP knockout (KO) cortical cells and *in vivo* in WT and STEP KO mice suggest specificity of inhibitors towards STEP compared to highly homologous tyrosine phosphatases. Furthermore, TC-2153 improved cognitive function in several cognitive tasks in 6- and 12-mo-old triple transgenic AD (3xTg-AD) mice, with no change in beta amyloid and phospho-tau levels.

## Abstract 2

This study identifies an unusual sulfur-based chemical as a novel and specific inhibitor of the tyrosine phosphatase STEP and shows that it can improve the cognitive function of a mouse model of Alzheimer's disease.

## Author Summary

A series of recent studies have found that the levels of the enzyme striatal-enriched protein tyrosine phosphatase (STEP) are raised in several different neuropsychiatric and neurodegenerative disorders, including Alzheimer's disease, fragile X syndrome, and schizophrenia. STEP normally opposes the development of synaptic strengthening, and these abnormally high levels of active STEP disrupt synaptic function by removing phosphate groups from a number of proteins, including several glutamate receptors and kinases. Dephosphorylation results in internalization of the glutamate receptors and inactivation of the kinases—events that disrupt the consolidation of memories. Here we identify the benzopentathiepin 8-(trifluoromethyl)-1,2,3,4,5-benzopentathiepin-6-amine hydrochloride (known as TC-2153) as a novel inhibitor of STEP. We show that the mechanism of action involves the formation of a reversible covalent bond between the inhibitor and the catalytic cysteine residue of STEP, and we demonstrate the activity of TC-2153 both *in vitro* and *in vivo*. TC-2153 shows specificity towards STEP compared to several other tyrosine phosphatases and shows no toxicity to cultured neurons. Importantly, the compound reversed cognitive deficits in a mouse model of Alzheimer's disease in a way that did not involve changes in the usual pathological signs (p-tau and beta-amyloid).

## Introduction

STriatal-Enriched protein tyrosine Phosphatase (STEP) (*PTPN5*) is a brain-enriched protein tyrosine phosphatase (PTP) targeted in part to postsynaptic terminals of excitatory glutamatergic synapses [1]–[4]. Recent studies indicate that STEP is overactive in Alzheimer's disease (AD), schizophrenia, and fragile X syndrome (FXS) [5]–[9]. The emergent model based on these findings suggests that the increase in STEP activity interferes with synaptic strengthening and contributes to the characteristic cognitive and behavioral deficits present in these disorders.

Elevated levels of STEP activity disrupt synaptic function by dephosphorylation of STEP substrates [10]. These include mitogen-activated protein kinase (MAPK) family members ERK1/2 and p38 [11],[12], the tyrosine kinases Fyn and Pyk2 [13],[14], the glutamate receptor GluN2B subunit of NMDARs (formerly termed NR2B) [6],[15],[16], and the GluA2 subunit of AMPAR (formerly termed GluR2) [16]–[18]. STEP dephosphorylates the kinases at regulatory tyrosine residues within their activation loop and thereby inactivates them. Dephosphorylation of GluN2B promotes internalization of GluN1/GluN2B receptors, whereas dephosphorylation of GluA2 promotes internalization of GluA1/GluA2 receptors.

To test the hypothesis that the observed overexpression of STEP disrupts synaptic strengthening in AD, we crossed STEP KO mice with the 3xTg-AD and Tg2576 AD mouse models. Six-month-old progeny null for STEP displayed significant decreases in biochemical and cognitive deficits, despite continued elevated levels of Aβ [16],[19]. These data validated STEP as a target for drug discovery. Herein we describe the search for small-molecule STEP inhibitors. We performed a high throughput screen that culminated in the identification of the benzopentathiepin 8-(trifluoromethyl)-1,2,3,4,5-benzopentathiepin-6-amine hydrochloride (known as TC-2153) as a novel STEP inhibitor. TC-2153 exhibited specificity for STEP *in vitro*, in cell-based assays, and *in vivo*, and also reversed cognitive deficits in 6- and 12-mo-old 3xTg-AD mice.

## Results

### Initial High Throughput Screening for STEP Inhibitors

**Figure 1:**
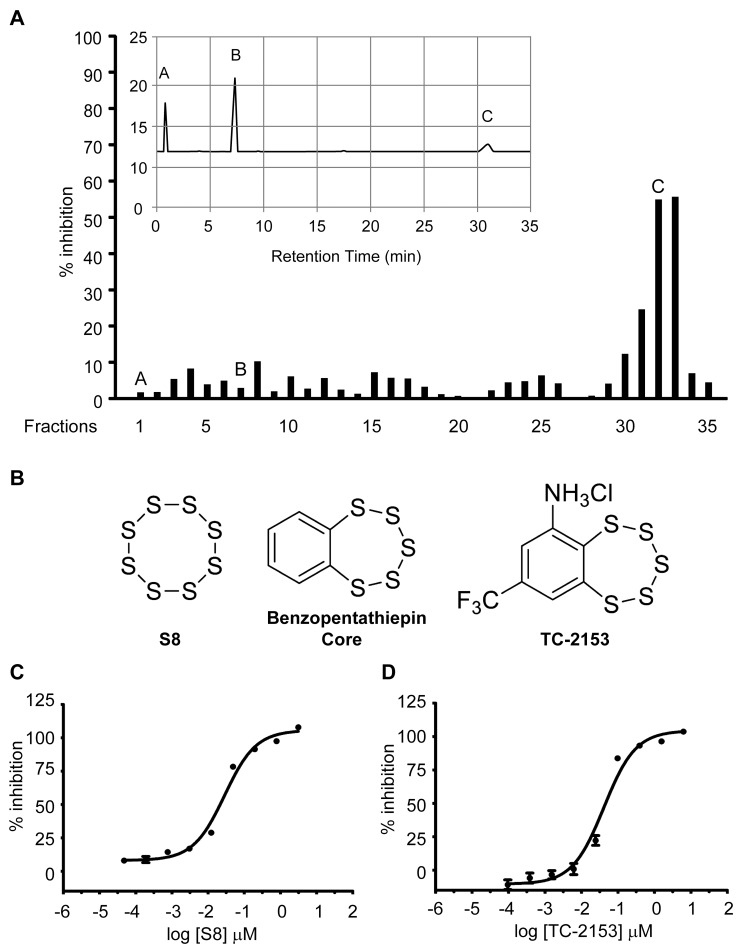
Compound 3 fractionation and initial characterization. (A) Commercially purchased Compound **3** was dissolved in methanol at 10 mg/mL, and 300 µL portions were injected onto a Zorbax (Agilent) 5 µm 300SB-C18 column (0.94×25 cm, 3 mL/min 75% methanol/25% pH 4.0 0.1 M ammonium acetate). Thirty-five fractions (3 mL each) were collected, evaporated, and reconstituted in 100 µL of DMSO. Fractions were tested with pNPP assays to determine inhibition of STEP activity by using 0.1 µL of each fraction and 100 nM of STEP protein in 96-well plates. DMSO alone was used as a control. Shown in the insert is a representative chromatogram (UV absorbance detection, 350 nm). Peaks A, B, and C indicate early unretained material, Compound 3, and the unknown compound. (B) Structure of S_8_, the benzopentathiepin core, and 8-(trifluoromethyl)-1,2,3,4,5-benzopentathiepin-6-amine hydrochloride (known as TC-2153). (C and D) Dose–response curves for S_8_ and TC-2153. (C) The IC_50_ for S_8_ was determined to be 17.2±0.4 nM (mean ± s.e.m., *n* = 4). (D) The IC_50_ for TC-2153 was determined to be 24.6±0.8 nM (mean ± s.e.m., *n* = 4).

We initially screened ∼150,000 compounds from the Laboratory for Drug Discovery in Neurodegeneration library using *para*-nitrophenyl phosphate (pNPP) as the target substrate (see Text S1 for more information on assay development and secondary screens). Eight compounds were selected for further characterization based on chemical structure and IC_50_ values, which ranged between 1 µM and 9.7 µM (Table S1), and studies of these molecules indicated potent inhibition of STEP activity in neuronal cultures and cortical tissue after intraperitoneal (i.p.) injections in WT mice. However, following resynthesis of several of the lead compounds, we found that they all exhibited essentially no inhibitory activity towards STEP (Figure S1). We therefore tested the possibility that a “contaminant” in the commercial preparations of the lead compounds was inhibiting STEP activity. To address this issue, we performed preparative HPLC on Compound **3** and tested eluted fractions for activity against STEP in the pNPP assay (Figure 1A). Compound **3** appeared as a major peak (fraction 7) on the reverse-phase HPLC preparation and had no inhibitory activity against STEP compared to a second peak that appeared as a late minor peak (fraction 32) that was a potent inhibitor of STEP. Given the high apparent lipophilicity of the unknown, the supplied material was extracted with hexane and recrystallized from methanol. Small pale yellow needle-shaped crystals (0.5–1 cm in length) were obtained in approximately 1% yield. The isolated crystalline material displayed the same HPLC retention, UV absorbance, and STEP inhibitory properties as the initially collected late-eluting peak. The crystalline compound was characterized by X-ray crystallography and found to be sulfur (S_8_) (Figure 1B).

S_8_ is poorly soluble in aqueous solution and cannot easily be modified to improve physicochemical properties, redox activity, binding affinity, and selectivity. We therefore sought to identify more conventional inhibitor structures that would improve solubility and enable further refinement through analog preparation and evaluation. We identified the benzopentathiepin core structure present in a number of natural products as the most promising for further investigation (Figure 1B). Natural products incorporating the benzopentathiepin core motif have been reported to have antifungal and antibacterial activity in cell culture as well as cytotoxicity against human cancer cell lines [19],[20]. Moreover, amino-substituted derivatives such as varacin and TC-2153 have reasonable solubility in aqueous solution [21],[22]. TC-2153 reportedly has a low level of acute toxicity (LD_50_>1,000 mg/kg) and was proposed to cross the blood brain barrier as evidenced by anxiolytic and anticonvulsant effects in mice [23]. We therefore chose to evaluate the STEP inhibitory activity of TC-2153. We first compared the inhibitory activities of S_8_ and TC-2153 against recombinant STEP using pNPP assays at several concentrations of the inhibitors. Both S_8_ and TC-2153 inhibited STEP potently, with IC_50_s of 17.2±0.4 nM and 24.6±0.8 nM, respectively (Figure 1C–D).

### STEP Inhibition Increases the Tyr Phosphorylation of STEP Substrates in Cortical Neurons and *in Vivo*

**Figure 2:**
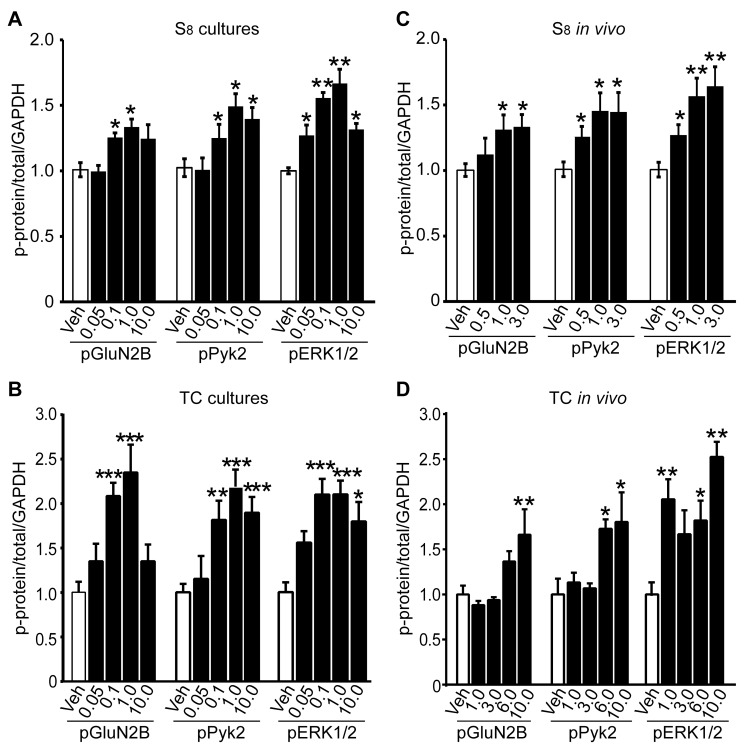
S_8_ and TC-2153 increases the Tyr phosphorylation of STEP substrates in neuronal cultures and *in vivo*. Cortical neuronal cultures were treated with (A) S_8_ and vehicle (Veh) or (B) TC-2153 and vehicle (0.05, 0.1, 1, and 10 µM) for 1 h. Phosphorylation of GluN2B (Y^1472^), Pyk2 (Y^402^), and ERK1/2 (Y^204/187^) were significantly higher after treatment of cultures with S_8_ (A) or TC-2153 (B) (*p<0.05, **p<0.01, ***p<0.001, one-way ANOVA with post hoc Bonferroni test). Data represent the phospho-signal normalized to total protein and then to GAPDH (mean ± s.e.m., *n* = 4). C57BL/6 mice (3–6 mo) were injected with (C) S_8_ (0.5, 1, 3 mg/kg, i.p.) or (D) TC-2153 (i.p., 1, 3, 6, 10 mg/kg, i.p.) and were sacrificed 3 h later. Cortices were microdissected and lysates spun down to P2 fraction and prepared for Western blotting. Tyrosine phosphorylation status was probed with phospho-specific antibodies to pGluN2B: Tyr^1472^, pPyk2: Tyr^402^, and pERK1/2: Tyr^204/187^ (*p<0.05; **p<0.01; one-way ANOVA with post hoc Bonferroni test). Data represent the phospho-signal normalized to the total protein signal and then to GAPDH (mean ± s.e.m., *n* = 3).

We treated cortical neurons for 1 h with S_8_ or TC-2153 and determined the Tyr phosphorylation of residues that STEP dephosphorylates on GluN2B (Y^1472^), Pyk2 (Y^402^), and ERK1/2 (Y^204/187^). For S_8_, there was a significant increase in the Tyr phosphorylation of all three STEP substrates at doses above 0.05 µM, with 1 µM showing maximum inhibition (Figure 2A and Figure S2A for representative blots) (1 µM dose, pGluN2B, 1.33±0.08, *p*<0.05; pPyk2, 1.49±0.12, *p*<0.05; pERK1/2, 1.67±0.14, *p*<0.01). For TC-2153, there was also a significant increase in the Tyr phosphorylation at these sites (Figure 2B and Figure S2B for representative blots) (1 µM dose, pGluN2B, 2.07±0.15, *p*<0.001; pPyk2, 1.81±0.21, *p*<0.001; pERK1/2, 2.39±0.18, *p*<0.001). The decrease in Tyr phosphorylation in the presence of the highest dose of TC-2153 (10 µM) may be due to off-target effects on positive regulatory PTPs. We found similar inverted-U dose–response curves on Tyr phosphorylation of direct PTP targets in previous work with PTP inhibitors [24],[25].

We next tested whether S_8_ and TC-2153 inhibited STEP activity in WT mice *in vivo*. Six-month-old male mice (C57BL/6) were injected with vehicle or S_8_ (0.5, 1, 3 mg/kg, i.p.) and cortices were removed and processed 3 h postinjection. S_8_ led to a significant increase in the Tyr phosphorylation of GluN2B, Pyk2, and ERK1/2 (at 1 mg/kg, pGluN2B, 1.31±0.11, *p*<0.05; pPyk2, 1.46±0.14, *p*<0.05; pERK1/2, 1.57±0.13, *p*<0.05) (Figure 2C and Figure S2C for representative blots). Similar results were obtained with TC-2153 (1, 3, 6, 10 mg/kg) (at 10 mg/kg, pGluN2B, 1.66±0.28, *p*<0.01; pPyk2, 1.80±0.30, *p*<0.05; pERK1/2, 2.52±0.16, *p*<0.01) (Figure 2D and Figure S2D for representative blots). Together, these results demonstrate that both S_8_ and TC-2153 increase the Tyr phosphorylation of three STEP substrates in intact neurons in culture and *in vivo* in the cortex of WT mice.

### Specificity of TC-2153 Against Other PTPs *in Vitro*

In an attempt to evaluate possible selectivity of TC-2153, we performed activity assays using the catalytic domain of STEP, and the catalytic domains of two highly related PTPs: He-PTP and PTP-SL. TC-2153 showed no apparent selectivity among these PTPs. Several studies have shown that regions outside of the catalytic domain contribute to the susceptibility of PTPs to the action of selective inhibitors [26],[27]. Thus, we repeated our assays using several full-length PTPs (Table 1). Indeed, we found TC-2153 was more potent against the two major isoforms of STEP, STEP_61_ (IC_50_ = 93.3±1.1 nM) and STEP_46_ (IC_50_ = 57.3±1.1 nM), compared to HePTP (IC_50_ = 363.5±1.2 nM) and PTP-SL (IC_50_ = 220.6±1.3 nM). It displayed even greater selectivity over PTP1B (IC_50_ = 723.9±1.2 nM) and SHP-2 (IC_50_ = 6896.0±1.2 nM). These results suggest TC-2153 shows a degree of selectivity toward full-length STEP in *in vitro* assays.

**Table 1:** Selectivity of TC-2153 *in Vitro*.

PTP	Accession^a^	IC_50_ (nM)^b^
STEP_61_	NP_001265167	93.3±1.1
STEP_46_	[62]	57.3±1.1
HePTP	AAH01746	363.5±1.2
PTP-SL	NP_002840	220.6±1.3
PTP1B	NP_002818	723.9±1.2
SHP-2	AAA36610	6,896.0±1.2

a from NCBI database; ^b^calculated using GraphPad Prism 5. mean ± s.e.m. (*n* = 3).

### TC-2153 Shows No Apparent Off-Target Effects in STEP KO Cultures

**Figure 3:**
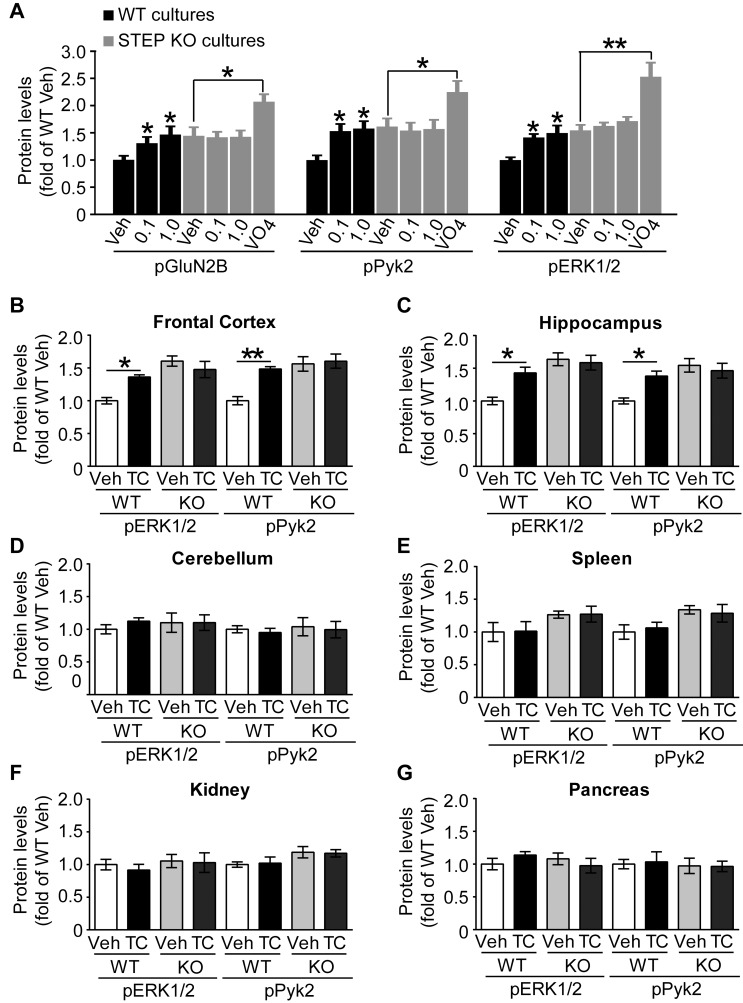
TC-2153 selectively inhibits STEP. (A) TC-2153 failed to increase tyrosine phosphorylation of STEP substrates in STEP KO cortical neurons. WT and STEP KO cultures were treated with TC-2153 (0.1 and 1 µM), vehicle (0.1% DMSO), or sodium orthovanadate (Na_3_VO_4_, 1 mM) for 1 h. Phosphorylation of GluN2B Y^1472^, Pyk2 Y^402^, and ERK1/2 Y^204/187^ was normalized to total protein level and then to GAPDH as loading control (*p<0.05, **p<0.01 one-way ANOVA with post hoc Bonferroni test, compared with veh-treated controls, *n* = 4). (B–G) TC-2153 increased the phosphorylation of ERK1/2 Y^204/187^ and Pyk2 Y^402^ in frontal cortex and hippocampus, but not in cerebellum, spleen, kidney, or pancreas, all tissues that do not have STEP. Mice were injected i.p. with TC-2153 (10 mg/kg; *n* = 4) or vehicle (n = 4) and were sacrificed 3 h later. Changes are expressed as the mean ± s.e.m. of pERK1/2 and pPyk2 normalized to total protein level and then to GAPDH (*p<0.05, **p<0.01; two-way ANOVA follow by Tukey's H.S.D. test).

To further address possible off-target inhibition by TC-2153 in cells, cortical cultures from either WT or STEP KO mice were treated with TC-2153. Similar to the rat neuronal cultures, we observed an increase in the Tyr phosphorylation of STEP substrates in WT mouse cortical neurons (Figure 3A, black bars). Consistent with previous findings [12],[16],[18],[28], STEP substrates have higher basal Tyr phosphorylation levels in STEP KO cultures. TC-2153 failed to increase the phosphorylation of STEP substrates in the KO cultures (Figure 3A, grey bar with 0.1 µM and 1 µM; see Figure S3A for representative blots). To exclude a possible ceiling effect, we added a generic tyrosine phosphatase inhibitor, sodium orthovanadate (Na_3_VO_4_), which further increased the Tyr phosphorylation of these substrates. These results suggest that TC-2153 is relatively specific towards STEP compared to the generic tyrosine phosphatase inhibitor sodium orthovanadate.

### TC-2153 does Not Inhibit Highly Homologous PTPs *in Vivo*

There are three highly related PTPs (STEP, HePTP, and PTP-SL) that all dephosphorylate ERK1/2. Only STEP is found in cortex, whereas HePTP is present in spleen, and PTP-SL is present in cerebellum, both tissues that lack STEP. In addition, ERK1/2 and Pyk2 are dephosphorylated by other tyrosine phosphatases outside of the CNS. We examined the specificity of TC-2153 by injecting WT and STEP KO mice with TC-2153 or vehicle, and determined the Tyr phosphorylation of ERK1/2 (Y^204/187^) and Pyk2 (Y^402^) in different organs (Figure 3B–G and Figure S3B for representative blots). There was a significant increase in pERK1/2 and pPyk2 in the frontal cortex and hippocampus, but not in the cerebellum or in all tissues tested outside the brain. These results suggest that TC-2153 does not target homologous PTPs known to dephosphorylate ERK1/2 and Pyk2 when tested *in vivo*.

**Figure 4:**
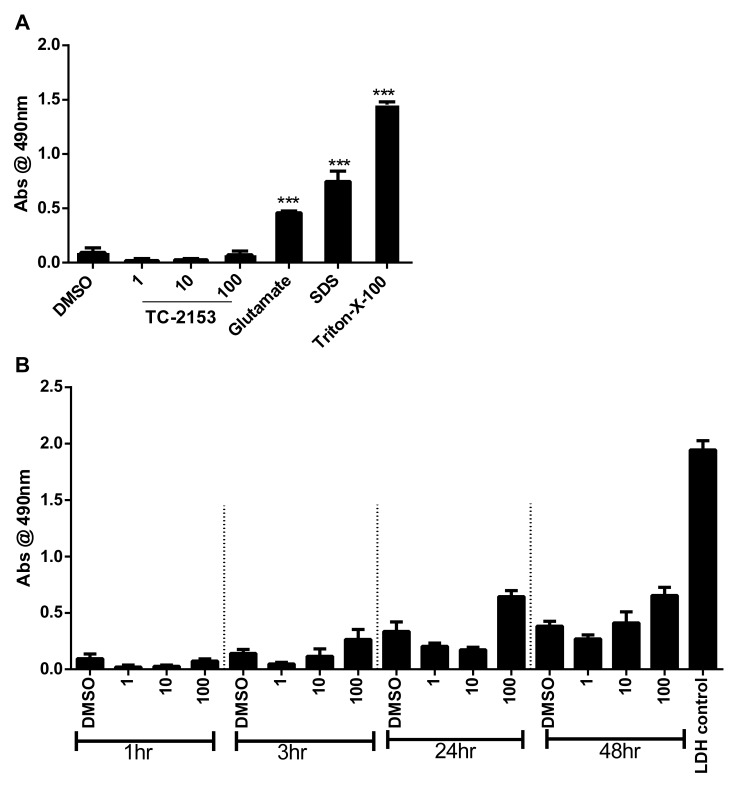
TC-2153 does not induce neuronal cell death. Cortical cells were incubated with TC-2153 (1, 10, and 100 µM) for 1 h along with positive controls: glutamate (100 µM), SDS (0.02%), and Triton-X-100 (0.15%) (A), and at multiple time points (1 h, 3 h, 24 h, and 48 h) (B). Bovine LDH was used as a LDH-positive control. The media was collected and analyzed for LDH. The assay quantitatively measures LDH, a stable cytosolic enzyme that is released upon cell lysis. Released LDH in culture supernatants is measured with a 30-min coupled enzymatic assay, which results in the conversion of a tetrazolium salt (INT) into a red formazan product. The amount of color formed is proportional to the number of lysed cells.

We also performed toxicity studies with TC-2153 in cortical cultures (Figure 4). We measured the release of lactate dehydrogenase (LDH) from the cultures for up to 48 h at various TC-2153 concentrations. Even at the high dose of 100 µM, TC-2153 had no significant effect on LDH release compared to the positive controls.

### Mechanism of STEP Inhibition by TC-2153

**Figure 5:**
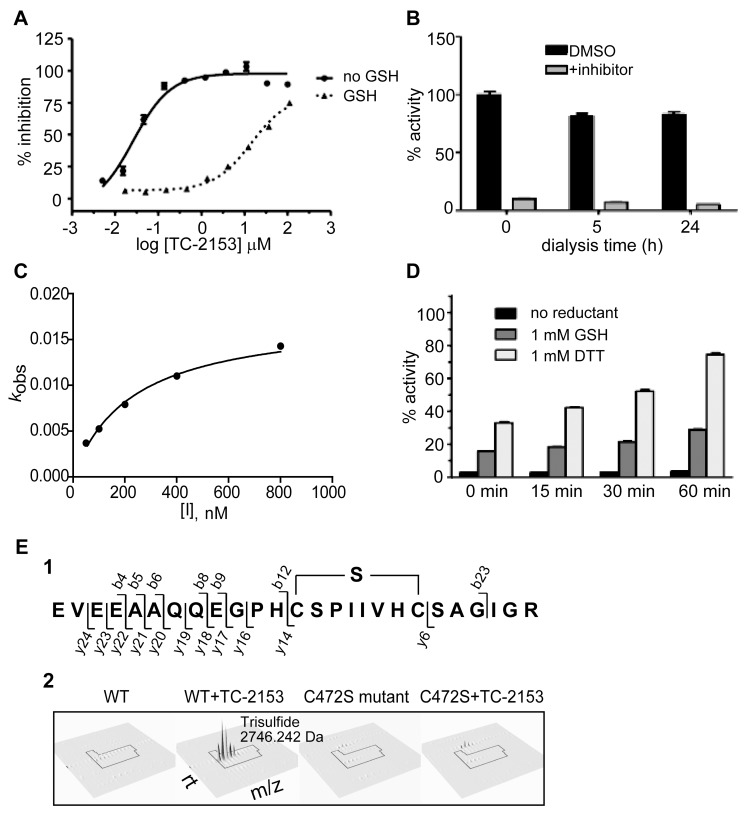
TC-2153 targets the active site cysteine of STEP. (A) STEP activity was measured with pNPP and IC_50_s were 24.6±0.8 nM and 8.79±0.43 µM in the absence and presence of 1 mM GSH (mean ± s.e.m., *n* = 2). (B) STEP (200 nM) and TC-2153 (1 µM) (or DMSO control) were incubated for 60 min to inhibit enzymatic activity prior to dialysis. Aliquots were tested against pNPP (mean ± s.e.m., *n* = 4). (C) The progress curve method was used to determine the second-order rate constant: *k*_inact_/*K*_i_ = 153,000±15,000 M^−1^s^−1^ (mean ± s.e.m., *n* = 4). (D) STEP (200 nM) and TC-2153 (5 µM) were incubated for 10 min and then incubated with GSH or DTT (1 mM each) or water (no reductant) for 0, 15, 30, or 60 min, and the enzymatic activity of STEP was measured using the pNPP assay (mean ± s.e.m., *n* = 4). (E) Detection of trisulfide bridge formation between C^465^ and C^472^. The peptide sequence in (1) illustrates the trisulfide bridge along with the b and y-ion assignments detected in the MS/MS fragmentations spectrum (see Figure S6). (2) compares the 3D elution profile of the trisulfide peptide (mass  = 2,746.242 Da). The trisulfide bridge (modified) peptide is only detected in the WT STEP in the presence of TC-2153. The corresponding disulfide (non-modified) peptide (mass  = 2,714.254 Da) was detected in WT STEP (see Figure S6).

We next examined the mechanism by which TC-2153 inhibited STEP. Because the catalytic cysteine in PTPs is prone to sulfhydration, nitrosylation, and oxidative modifications that cause inhibition of phosphatase activity [27]–[32], we first examined the effect of a reducing agent on STEP inhibition by TC-2153. The addition of reduced glutathione (GSH, 1 mM) decreased the inhibitory activity of TC-2153 by two orders of magnitude in *in vitro* assays (IC_50_ = 8.79±0.43 µM compared to 24.6±0.8 nM) (Figure 5A). These results suggested an oxidative mechanism for the inhibition of STEP. We established that TC-2153 was stable and did not degenerate in the assay conditions by sensitive ^19^F NMR monitoring (Figure S4) and was not acting through generation of reactive oxygen species (ROS), which was tested by the addition of catalase or superoxide dismutase to the *in vitro* assay (Table S2). To confirm that ROS are also not released in cortical cultures with TC-2153 treatment, we performed H_2_O_2_ colorimetric assay and fluorescence assay with 2,7-dichlorofluorescein diacetate (DCF) and did not observe any significant differences in H_2_O_2_ or ROS levels between the TC-2153 treated compared to nontreated control groups (Figure S5).

To evaluate the mode of inhibition, we incubated STEP with TC-2153, subjected the sample to dialysis to remove excess inhibitor, and monitored enzyme activity (Figure 5B). After 24 h of dialysis, STEP remained inhibited, suggesting that TC-2153 acts as an irreversible inhibitor under the conditions used. Using the progress curve method [29], inhibition was also found to be irreversible and the second order rate of inactivation was determined (Figure 5C). A *k*_obs_ was determined for pNPP in the presence of varying initial inhibitor concentrations (*n*≥4). Values were then analyzed with nonlinear regression to obtain the kinetic constants: *k*_inact_ = 0.0176±0.0007 s^−1^; *K*_i_ = 115±10 nM; *k*_inact_/*K*_i_ = 153,000±15,000 M^−1^s^−1^. However, STEP activity could be recovered following incubation with GSH or DTT (Figure 5D). Aliquots of STEP were incubated with DMSO control or TC-2153 and were then added to assay buffer containing 1 mM GSH, 1 mM DTT, or water control and allowed to incubate for up to 1 h prior to testing for enzymatic activity. STEP activity was rapidly recovered by both reductants, with DTT showing a greater recovery of activity (75% recovery after 1 h, where DMSO control represents 100% activity) compared to GSH (29% recovery after 1 h).

We then performed LCMS analysis to determine the intact protein mass of STEP and STEP+TC-2153. Our intact protein analyses suggest a covalent adduct to STEP. Although we were able to obtain the accurate mass for STEP, we were unable to mass spectrally resolve the heterogeneous mixture of intact STEP+TC-2153 and its covalent adducts with sufficient accuracy to fully interpret the results. Therefore, we next used high-resolution tandem mass spectrometry to focus upon whether TC-2153 might modify the active site cysteine of STEP. For these experiments, we used WT STEP as well as a STEP mutant in which the catalytic cysteine was changed to serine. Greater than 90% of the primary amino acid sequences were identified by LC-MS/MS for WT STEP or for the STEP mutant, following in-gel tryptic digestion of STEP from nondenaturing (native) preparations. We initially analyzed the catalytic cysteine at position 472 of STEP in the absence of TC-2153 and found a disulfide bridge between Cys^465^ and Cys^472^ that presumably forms following tryptic digestion given the positions of Cys^465^ and Cys^472^ in the three-dimensional X-ray crystal structure of STEP [30]. This modification was not observed when the catalytic site cysteine (Cys^472^) was mutated to serine. Incubation of WT STEP with TC-2153 resulted in the presence of a *de novo* trisulfide within the Cys^465^/Cys^472^ bridge, which was not observed for WT STEP alone or when the catalytic site cysteine (Cys^472^) was mutated to serine (Figure 5E and Figure S6). The precursor monoisotopic mass of the trisulfide-containing peptide had a mass error of 4 ppm (∼0.011 Da) based on theoretical mass calculation, which is within the 5 ppm external mass calibration expected for MS/MS data collected by the linear ion trap instrument used. These results indicate that the active site cysteine is likely modified by TC-2153 and suggest that following tryptic digestion a sulfur from the benzopentathiepin core is retained, giving rise to the trisulfide identified by mass spectrometry.

### TC-2153 Reduces Cognitive Deficits in 3xTg-AD Mice

**Figure 6:**
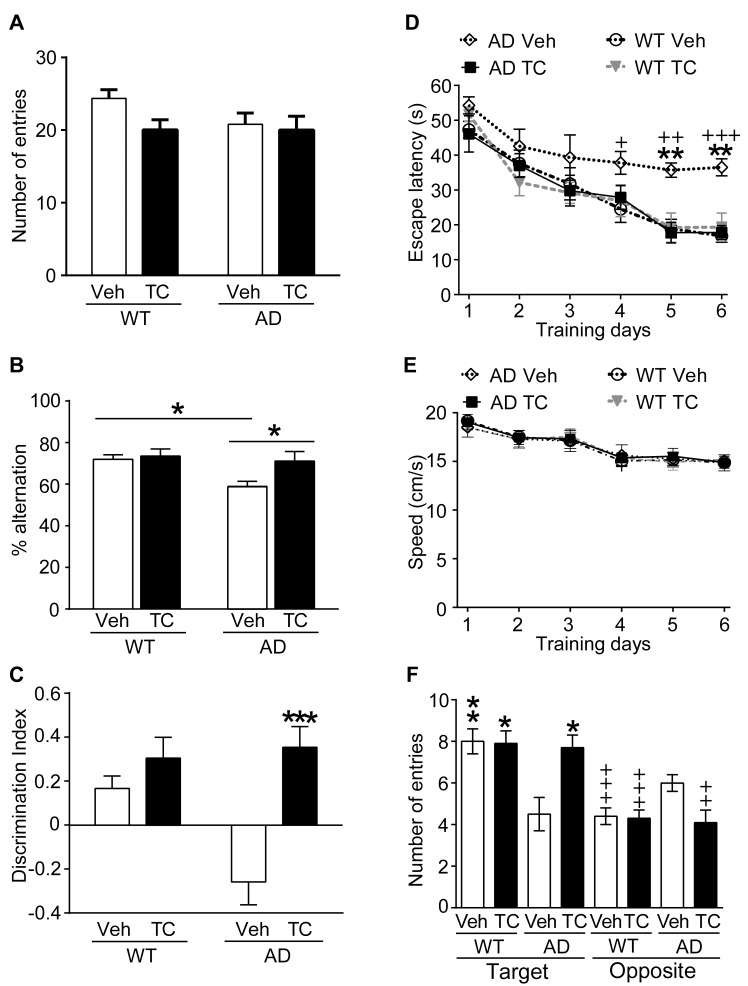
TC-2153 improves cognitive deficits in 3xTg-AD mice. WT and 3xTg-AD mice (male, 6 mo old) were treated with vehicle or TC-2153 (10 mg/kg, i.p.) and tested in the Y-maze, NOR, and MWM tasks. (A and B) Y-maze, number of arm entries and percentage spontaneous alternations were calculated (*p<0.05, paired *t* test, AD-TC versus AD-Veh) (WT, *n* = 20/group; AD, *n* = 11/group). (C) NOR, the DI of each group was calculated (***p<0.001, AD-TC versus AD-Veh) (WT, *n* = 9/group; AD, *n* = 16/group). (D) MWM, the 3xTg-AD mice injected with vehicle (*n* = 6) showed longer escape latency before finding the hidden platform (3 trials/day; 60 s; 30 m intertrial interval) when compared to AD mice treated with TC-2153 (*n* = 7) or WT mice injected with vehicle (*n* = 12) or TC-2153 (*n* = 13) (three-way ANOVA). * and ^+^ represents a statistical significant variation between AD-Veh mice and AD-TC or WT-Veh, respectively. (E) Swim speed at each training day was not significantly different between groups (three-way ANOVA). (F) Number of entries in a circular zone positioned around the previous platform location and in the opposite quadrants. * represents a statistical significant variation between AD-TC mice and other groups for the target quadrant. ^+^ indicates a difference for the target and opposite quadrant within each group. Data are mean ± s.e.m. *,^+^ *p*<0.05; **,^++^ *p*<0.01; ***,^+++^ *p*<0.01.

We next tested the efficacy of TC-2153 to reverse cognitive deficits in an AD mouse model. We first used the Y-maze to evaluate spatial working memory function in 3xTg-AD mice. AD or WT mice were injected with vehicle or TC-2153 (10 mg/kg, i.p.) 3 h prior to the test. Spontaneous alternations and total arm entries were calculated. There was no significant change in arm entries in TC-2153–treated mice, suggesting no drug-induced effect on general motor activity (Figure 6A). The main effect of genotype [*F*(1, 29) = 5.240, *p*<0.05], treatment [*F*(1, 29) = 5.895, *p*<0.05], and Genotype × Treatment interaction [*F*(1, 29) = 9.751, *p*<0.01] were significant for spontaneous alternations, with the AD mice making more incorrect choices (i.e., fewer alternations) (Figure 6B). Compared to their respective vehicle controls, TC-2153 increased percentage alternation in the AD mice (TC-treated, 71.13±4.58 versus Veh-treated, 58.94±2.46, *p*<0.05), but not in WT mice (TC-treated, 73.45±3.19; Veh-treated, 74.98±2.19, *p*>0.05).

We next investigated whether TC-2153 improved performance in the novel object recognition (NOR) task. WT or 3xTg-AD mice had no significant differences in baseline locomotor activity as measured during the habituation phase. Mice received an acute injection of vehicle or TC-2153 (10 mg/kg) 3 h prior to the training phase. Twenty-four h later, mice were subjected to the test phase. Discrimination indexes (DIs) were compared for group differences in object memory. The main effect of genotype [*F*(1, 23) = 4.342, *p*<0.05], treatment [*F*(1, 23) = 5.895, *p*<0.01], and Genotype × Treatment interaction was significant [*F*(1, 23) = 4.362, *p*<0.05]. Post hoc analysis indicated that the DI in the AD-TC group was significantly higher than those of the AD-Veh group (TC-treated, 0.354±0.094 versus vehicle-treated, −0.259±0.104, *p*<0.001). In the WT groups, the DI in the TC-2153–treated mice did not differ from the Veh-treated mice (vehicle-treated, 0.166±0.057; TC-treated, 0.304±0.095, *p*>0.05) (Figure 6C).

We then tested the effects of TC-2153 in the reference memory version of the Morris water maze (MWM). A three-way ANOVA analysis revealed a significant Genotype × Treatment × Training Day interaction (*p*<0.05). Daily injection of TC-2153 3 h prior to training reversed memory deficits in 3xTg-AD mice on days 5 and 6 of the acquisition phase (*p*<0.01) (Figure 6D). The longer escape latency of 3xTg-AD mice injected with vehicle was not attributed to slower swimming speed, as no significant differences were found between groups (*p*>0.05; two-way ANOVA) (Figure 6E). To confirm memory status, the number of entries in a circular zone located around the previous platform location (target zone) and in the opposite quadrants was evaluated during the probe trial 24 h after the last acquisition day. A three-way ANOVA analysis revealed a significant Genotype × Treatment × Quadrant interaction (*p*<0.004). The 3xTg-AD mice treated with TC-2153 spent as much time as WT mice in the target zone, whereas AD mice injected with vehicle showed no preference for the target zone (Figure 6F). All groups had similar escape latencies during the cued trial when the platform was visible, indicating the absence of sensorimotor or motivational deficits to escape from water (WT-Veh, 15.1±1.7 s; WT-TC, 15.6±1.7 s; AD-Veh, 15.3±3.0 s; AD-TC, 16.0±2.3 s; mean ± s.e.m.; *p*>0.05; two-way ANOVA). There were no differences in thigmotaxic swimming patterns between any of the tested groups (Figure S7). Taken together, these results demonstrate that TC-2153 significantly improved cognitive functioning in 6-mo-old 3xTg-AD mice.

We next determined whether inhibition of STEP in 12-mo-old 3xTg-AD mice affected beta amyloid or phospho-tau levels. We first needed to confirm that TC-2153 was effective in attenuating cognitive deficits at 12 mo, as these mice have more robust increases in phospho-tau and Aβ levels. We tested the mice with the NOR task and once again found a significant improvement of memory in AD mice treated with TC-2153 during the choice phase (10 mg/kg, i.p.; TC-2153-AD, familiar versus novel, *p*<0.05). TC-2153 did not affect cognitive function in WT mice (Figure S8A). There were no significant changes in Aβ or phospho-tau levels after administration of TC-2153 (Figure S8B–C).

## Discussion

STEP function is disrupted in several neurological disorders in addition to AD, including FXS [9], Parkinson's disease [31], and schizophrenia [8]. The increase in STEP expression in these illnesses is due to either an increase in its translation (in FXS) or a decrease in its degradation (in AD, Parkinson's disease, and schizophrenia). In contrast, STEP levels or activity are lower in several other disorders, including stress-related conditions [32],[33], excessive EtOH consumption [34], and cerebral ischemia [35]. Thus, the current model is that STEP activity must be within an optimal range and that either high or low levels of STEP disrupt synaptic plasticity. Disruption in STEP function has also been implicated in seizures [36], ethanol abuse [37], amphetamamine-induced stereotypies [38], and Huntington's disease [39],[40], although the basis for these changes remain to be determined.

In terms of STEP dysfunction, most is known about its role in AD. Aβ binding to the α7 nicotinic receptor leads to calcium influx and calcineurin activation [6]. Calcineurin activates protein phosphatase 1 (PP1), which dephosphorylates a regulatory serine residue within the STEP substrate-binding domain, enabling STEP to interact with and dephosphorylate its substrates [41]. In addition, STEP is normally ubiquitinated and degraded by the proteasome after NMDAR stimulation [15], and Aβ inhibition of the proteasome [42],[43] results in a build-up of active STEP [7]. Based on these results, STEP was genetically lowered by crossing STEP KO mice with triple transgenic mice to produce progeny that still had the AD mutations, but were null for STEP [16]. These progeny had improved cognitive function and led to the current study to discover STEP inhibitors.

We initially developed an HTS assay for STEP using the generic phosphatase substrate pNPP and screened ∼150,000 compounds to identify STEP inhibitors. However, when lead compounds were resynthesized, they had significantly lower activity against STEP, suggesting that an impurity in the commercial substances was likely inhibiting STEP activity. We isolated and identified this “contaminating” compound as elemental sulfur in the form of octasulfur (S_8_), which led in turn to the identification of the lead compound TC-2153. It should be noted that we did not include the reductant DTT in the initial library screen, in contrast to many screens for PTP inhibitors [44]. This allowed us to discover S_8_ and identify TC-2153 as a potent inhibitor of STEP and the mechanism of action by which TC-2153 inhibits STEP.

The specificity of TC-2153 in *in vitro*, as well as cell and animal models, was explored. Interestingly, TC-2153 was more selective in *in vitro* assays against full-length STEP, but showed little specificity when tested against the truncated phosphatase domains of the PTPs. Although the exact mechanisms need to be clarified, these results are consistent with other recent findings with PTP inhibitors [45],[46].

TC-2153 treatment of neuronal cultures and WT mice increased the Tyr phosphorylation of STEP substrates. These results are consistent with previous studies of STEP KO mice that showed that a loss of STEP results in an increase in the Tyr phosphorylation of all STEP substrates identified to date [10],[12],[14],[28]. The Tyr phosphorylation of the three STEP substrates tested was not significantly changed by TC-2153 in STEP KO neuronal cultures, but was increased by the general PTP inhibitor sodium orthovanadate, indicating that a ceiling effect did not explain the results.

Similarly, administration of TC-2153 to WT mice led to an increase in the Tyr phosphorylation of the STEP substrates ERK1/2 and Pyk2 in the hippocampus and frontal cortex (where STEP is present). However, treatment of WT mice with TC-2153 did not increase the Tyr phosphorylation of these two substrates in regions with no STEP expression (i.e., the cerebellum and organs outside of the CNS). An important finding was that treatment of STEP KO mice did not increase the Tyr phosphorylation of these substrates over baseline levels in the hippocampus and frontal cortex. The Tyr phosphorylation of ERK1/2 and Pyk2 did not alter in the cerebellum or in organs outside of the CNS (regions were STEP is not expressed). These results suggest a significant degree of *in vivo* specificity for inhibition of STEP by TC-2153, although additional studies are needed to expand on these initial findings.

These results are consistent with an emerging body of research that suggests that oxidative regulation of the catalytic cysteine residue of PTPs is an important regulatory mechanism *in vivo* that links tyrosine phosphorylation signaling and the redox status of cells [47],[48]. PTPs contain a catalytic cysteine with an SH-group that exists in a thiolate state (S^–^) and facilitates removal of phosphate groups from substrates. The pKa values of these cysteine residues are in the range of 4–6, making these sites more likely to be oxidized compared to other cysteines that typically have pKa values in the range of 8–9 [49],[50]. Thus, a high reducing environment in cells, either through reduced production of reactive oxygen species or elevated activity of reactive oxygen scavengers, is proposed to decrease PTP oxidation and increase PTP activity. In contrast, a high oxidizing environment would increase PTP oxidation, reduce PTP activity, and increase tyrosine kinase signaling [49],[51],[52]. Although all PTPases are likely to modulate qualitatively in a similar fashion by cell redox status, several studies have shown that PTPs can be differentially inhibited by oxidation. For example, stimulation of T-cell receptors results in a selective oxidation of SHP2, but not SHP1, whereas both PTPs show similar sensitivity to oxidation *in vitro*. In contrast, SHP1 is more prone to oxidation than SHP2 after treatment of EOL-1 cells with H_2_O_2_ [26].

Whether STEP is regulated in cells by a similar balance of reactive oxygen species and oxygen scavengers, and whether this might explain the sensitivity of STEP to TC-2153, is under investigation. In the initial studies presented here, we explored the mechanism for STEP inhibition. Irreversible inhibition in the absence of reducing conditions was suggested for TC-2153, as dialysis did not restore activity. However, enzyme activity could be recovered by incubation with DTT or GSH, consistent with enzyme inactivation by oxidative modification of the active site cysteine. Moreover, the inhibitory activity of TC-2153 was considerably reduced by addition of DTT or GSH to the assay buffer. The oxidative mechanism for inactivation observed for TC-2153 is in agreement with established oxidative mechanisms for regulating PTP activity *in vivo* [53]–[55]. In support of this, mass spectroscopy analysis indicated that the catalytic Cys residue (Cys^472^) was modified by a trisulfide bridge that included Cys^465^, but only in the presence of TC-2153.

In summary, we have discovered that the pentathiepin TC-2153 potently inhibits STEP activity. Although the results from the STEP KOs and the biochemistry are suggestive of a direct action of TC-2153 on the STEP active site, we cannot exclude an indirect mechanism *in vivo*. An important finding is that the new platform represented by TC-2153 reverses cognitive deficits in 6- and 12-mo-old 3xTg-AD mice, a reflection of the suggested role of STEP in the initial synaptopathology of AD [6],[16],[56]. The administration of TC-2351 did not affect the Aβ and tau brain pathology of the mice, although the question of the efficacy of TC-2153 at advanced stages of pathology remains open. Longitudinal studies with TC-2153 as well as administration of TC-2153 to other AD models will help address this question. Longitudinal studies will also address the long-term preventive effects of STEP inhibition on cognitive decline. Finally, it is important to determine whether TC-2153 is effective in other animal models of neuropsychiatric diseases in which STEP activity is elevated and these studies have begun.

## Materials and Methods

### Ethics Statement

The Yale University Institutional Animal Care and Use Committee approved all proposed use of animals. All animal work was carried out in strict accordance with National Institutes of Health (NIH) Guidelines for the Care and Use of Laboratory Animals.

### Reagents

pNPP, 2-(N-morpholino) ethanesulfonic acid (MES), sodium orthovanadate, ATP, and all buffer components were purchased from Sigma-Aldrich (St. Louis, MO). Malachite Green reagent kit was purchased from Bioassay system (Hayward, CA). 6,8-difluoro-4-methylumbelliferyl phosphate (DiFMUP) and EnzChek phosphatase assay kit were purchased from Invitrogen (Carlsbad, CA). The 96- and 384-well clear polypropylene plates were purchased from VWR (Radnor, PA), and 384-well white plates were purchased from Nalge Nunc International (Rochester, NY). Full-length STEP_46_ was used in the initial library screen. STEP_46_ cDNA was cloned into pGEX2T and transformed into BL21 (DE3) *E. coli* cells. STEP (20 mg) was purified on a glutathione sepharose column to immobilize the GST-tagged protein [14]. The column was loaded, washed, and bound protein eluted using Fast Protein Liquid Chromatography. For some of the biochemical experiments, we purified WT TAT-STEP_46_ and TAT-STEP_46_ (C to S) proteins, the latter containing a mutation at its catalytic cysteine within the active site that renders the enzyme inactive [15]. The assay development for the HTS is shown in Figure S9, biochemical characterization of STEP is in Figure S10, and the synthesis of TC-2153 (benzopentathiepin 8-(trifluoromethyl)-1,2,3,4,5-benzopentathiepin-6-amine hydrochloride) is described in detail in Figure S11.

### Cell-Based Assay

Primary cortical neurons were isolated from Sprague Dawley rat embryos (E18) (Charles River Laboratories, Wilmington, MA) as previously described [15]. In some experiments, cortical neurons were made from WT and STEP KO mouse embryos (E18). Neurons were allowed to grow for 18–21 d in CO_2_ incubator before addition of compounds at indicated doses for 1 h. Immediately following treatments, neurons were lysed in RadioImmuno Precipitation Assay (RIPA) buffer supplied with protease inhibitor cocktail (Roche Applied Science, Indianapolis, IN) and phosphatase inhibitors (NaF and Na_3_VO_4_). All experiments were replicated four times with four independent batches of cultures.

### In Vivo Work

Wild-type, male C57BL/6 mice (3–6 mo) were used for all studies. An initial dose–response curve was carried out using S_8_ (0.5, 1, and 3 mg/kg, i.p.) or TC-2153 (1, 3, 6, and 10 mg/kg, i.p.). Pilot studies were conducted to optimize the time after i.p. injection when STEP substrates showed maximum Tyr phosphorylation (1–3 h). Cortical tissues were dissected out 3 h postinjection and processed for subcellular fractionation. We homogenized brain tissue in buffer containing (in mM): 10 Tris-HCl, pH 7.6, 320 sucrose, 150 NaCl, 5 EDTA, 5 EGTA, 20 NaF, 1 Na_3_VO_4_, and protease inhibitors (TEVP). Homogenates were centrifuged at 800 × *g* to remove nuclei and large debris (P1). Synaptosomal fractions (P2) were prepared from S1 by centrifugation at 9,200 × *g* for 15 min. The P2 pellet was washed twice and was resuspended in TEVP buffer. In some experiments, mice were injected with S_8_ (1 mg/kg, i.p.) or TC-2153 (3 mg/kg, i.p.), and cortex, cerebellum, and spleen were removed to test for the *in vivo* inhibition of the highly related PTPs, HePTP, and PTP-SL [57]–[60].

### Western Blotting

Samples were prepared and resolved by SDS-PAGE, transferred to nitrocellulose membrane, and incubated with phospho-specific antibodies (anti-pY^204/187^ ERK1/2, anti-pY^402^ Pyk2, anti-pY^1472^ GluN2B) or total protein antibodies (anti-ERK2, anti-Pyk2, and anti-NR2B) overnight at 4°C. All antibodies used are listed in Table S3. Immunoreactivity was visualized using a Chemiluminescent substrate kit (Pierce Biotechnology, Rockford, IL) and detected using a G:BOX with the image program GeneSnap (Syngene, Cambridge, UK). All densitometric quantifications were performed using the Genetools program.

### Isolation and Identification of S_8_ as Active Constituent of Compound 3

Compound **3** was extracted with hexane and the residue obtained after rotary evaporation, then recrystallized from methanol. Small pale yellow needle-shaped crystals (0.5–1 cm) were obtained in approximately 1% yield. The isolated crystalline material displayed the same HPLC retention, UV absorbance, and STEP inhibitory properties as the initially collected late-eluting peak. The crystalline compound was characterized by the X-Ray Crystallographic Facility of the Yale University Department of Chemistry and found to be sulfur (S_8_).

### General Procedures for Determination of Inhibitor IC_50_

Reaction volumes of 100 µL were used in 96-well plates. We added 75 µL of water to each well, followed by 5 µL of 20× buffer (stock, 1 M imidazole HCl, pH 7.0, 1 M NaCl, 0.02% Triton-X 100). We added 5 µL of the appropriate inhibitor dilution in DMSO, followed by 5 µL of phosphatase (stock, 0.2 µM, 10 nM in assay). The assay plate was then incubated at 27°C for 10 min with shaking. The reaction was started by addition of 10 µL of 10× pNPP substrate (stock, 5 mM, 500 µM in assay), and reaction progress was immediately monitored at 405 nm at a temperature of 27°C. The initial rate data collected were used for determination of IC_50_ values. For IC_50_ determination, kinetic values were obtained directly from nonlinear regression of substrate–velocity curves in the presence of various concentrations of inhibitor using one site competition in GraphPad Prism v5.01 scientific graphing software. The K_m_ value of pNPP in this system was determined to be 745 µM and was used in the kinetic analysis.

For experiments with catalase or superoxide dismutase (SOD), 10 µL of the appropriate enzyme stocks (catalase, 800 U/mL stock, 80 U/mL in assay; SOD, 1,000 U/mL stock, 100 U/mL in assay) were added prior to addition of the inhibitor and STEP.

For the experiments with glutathione reducing agent, 10 µL of glutathione (stock, 10 mM, 1 mM in assay) or water control was added before the inhibitor stocks, and only 65 µL of water was added initially to maintain the 100 µL assay volume. Once the inhibitor stocks were added, the assay plate was allowed to incubate 10 min at 27°C with shaking. This was followed by addition of phosphatase (stock, 0.4 µM, 20 nM in assay) and another 10-min incubation at 27°C prior to addition of pNPP substrate.

### Selectivity of TC-2153 Against STEP *in Vitro*

Purification of GST-tagged STEP_61_ and STEP_46_ constructs was as previously described [13],[14]. The GST-SHP-2 construct was a generous gift from Dr. A. M. Bennett (Yale University). The GST-PTP1B construct was purchased from Addgene (Cambridge, MA). Constructs were transformed into BL21 (DE3) *E. coli* cells. Fusion proteins were purified on a glutathione sepharose column. Full-length HePTP and PTP-SL proteins were purchased from Abnova (Taipei, Taiwan). All proteins were dialyzed in 1,000-fold volume of buffer, which was repeated three times. Assays were carried out in 96-well plates with 10 nM of each phosphatase and various doses of TC-2153 in triplicates. After 10 min preincubation of enzyme and inhibitor at 27°C, 500 µM of pNPP was added and incubated for 30 min. Absorbance was taken at 405 nm using a Biotek plate reader. Percent of inhibition by TC-2153 at each dose was calculated and plotted using GraphPad Prism v5.01 to obtain IC_50_.

### Determination of TC-2153 Stability in Imidazole Buffer

To monitor the stability of TC-2153 in the imidazole buffer, 20 µL of 20 mM TC-2153 stock in DMSO was added to an Eppendorf tube. The solution was diluted to 400 µL (1 mM TC-2153 final concentration, 5% final DMSO) with either water or the pH 7.0 imidazole buffer. The tube was allowed to incubate at ambient temperature with shaking for 1 h. The mixture was diluted with 150 µL of DMSO-*d_6_* and transferred to an NMR tube containing a capillary of trifluoroacetic acid as an external standard (−76.55 ppm). The stability of the compound in the buffer was confirmed by observing no differences in the sensitive ^19^F-NMR spectra (Figure S4). As a control for compound modification, the experiment was repeated with the addition of 1 mM GSH in the incubation buffer.

### Dialysis

STEP was diluted into 1× assay buffer with either inhibitor or DMSO control (final volume, 2.9 mL; final concentration, 1 µM STEP, 5 µM TC-2153; 50 mM imidazole HCl, pH 7.0, 50 mM NaCl, 0.001% Triton-X 100, 5% v/v DMSO). The samples were shaken at room temperature for 1 h to inhibit STEP. Each sample was then transferred to a separate Thermo Scientific Slide-A-Lyzer dialysis cassette with a 10,000 MW cutoff and 0.5–3.0 mL sample volume and was dialyzed into 1 L of 1× assay buffer over 24 h in a 4°C cold room. Aliquots of approximately 100 µL were removed from the dialysis cassette at 0, 4, and 24 h time points. Protein concentration was determined by reading absorbance at 280 nm compared to a standard curve for STEP. The samples were diluted to 100 nM in 100 µL of 1× assay buffer. The reaction was started by addition of 10 µL of 10× pNPP substrate (stock, 20 mM, 1.81 mM in assay; total assay volume, 110 µL), and reaction progress was immediately monitored at 405 nm at a temperature of 27°C. The initial rate data collected were used to determine enzyme activity standardized to the DMSO control zero time point.

### Recovery of STEP Activity by Reducing Agents

STEP was diluted to 200 nM in water, and aliquots of this stock were mixed with DMSO (5% by volume) or TC-2153 (5 µM final concentration, 5% DMSO by volume) and incubated at ambient temperature on a shaker for 10 min. Each sample was aliquoted out and 50 µL was transferred to wells of a 96-well microtiter plate containing 40 µL of 2× assay buffer with added reductant (GSH or DTT, 1 mM final concentration) and shaken for 0, 15, 30, or 60 additional minutes at ambient temperature. The reaction was started by addition of 10 µL of 10× pNPP substrate (stock, 20 mM, 2 mM in assay; total assay volume, 100 µL), and reaction progress was immediately monitored at 405 nm at a temperature of 27°C. The initial rate data collected were used to determine enzyme activity standardized to the DMSO controls.

### Determination of Inhibition Constants

The second-order rate constant of inactivation for TC-2153 was determined under pseudo–first-order conditions using the progress curve method [29]. Assay wells contained a mixture of the inhibitor (800, 400, 200, 100, 50, 0 nM) and 745 µM of pNPP (K_m_ = 745 µM) in buffer (50 mM imidazole pH 7.0, 50 mM NaCl, 0.01% Triton-X 100). Aliquots of STEP were added to each well to initiate the assay. The final concentration of STEP was 10 nM. Hydrolysis of pNPP was monitored spectrophotometrically for 30 min at an absorbance wavelength of 405 nm. To determine the inhibition parameters, time points for which the control ([I] = 0) was linear were used. A *k*_obs_ was calculated for each inhibitor concentration via a nonlinear regression of the data according to the equation P  =  (*v*_i_/*k*_obs_)(1-exp(-*k*_obs_ *t*)) (where P, product formation; *v*_i_, initial rate; *t*, time) using Prism 5 (GraphPad). Because *k*_obs_ varied hyperbolically with [I], nonlinear regression was performed to determine the second-order rate constant, *k*_inact_/*K*_i_, using the equation *k*_obs_  =  *k*_inact_[I]/([I] + *K*_i_ (1 + [S]/ K_m_)). Assays were done in quadruplicate on two separate occasions. The average and standard deviation of the assays is reported.

### Mass Spectrometry

To explore the protein modification(s) of STEP upon TC-2153 inhibition, reduced and nonreduced gel-purified STEP (WT or C472S mutant) proteins were analyzed by high-resolution tandem mass spectrometry. Briefly, purified STEP WT or C-S mutant proteins (10 µg each) were incubated with vehicle (1% DMSO) or TC-2153 (10 µM in 1% DMSO) in assay buffer (50 mM imidazole, pH 7.0) at room temperature (25°C) for 30 min. Samples were resolved on 8% SDS-PAGE or nondenaturing PAGE, and proteins were visualized by Coomassie Blue staining. Gel bands were excised and kept at −80°C until use. Excised gel bands corresponding to the mutant and WT STEP with and without TC-2153 were in-gel trypsin digested under native conditions (w/o reducing agent) overnight. Peptides were extracted from the digested samples with 80% acetonitrile containing 0.1% trifluoroacetic acid, and then dried under SpeedVac. Samples were then reconstituted in minimum solution containing 0.1% TFA, and loaded onto a RP C18 nanoACQUITY UPLC column (1.7 µm BEH130 C18, 75 µm×250 mm, with a 5 µm Symmetry C18 2G-V/M Trap [180 µm×20 mm]). Eluted peptides were directly infused into an Orbitrap Elite LC MS/MS system running data-dependent acquisition. Acquired data were processed utilizing Progenesis LCMS software (Nonlinear Dynamics) and MASCOT Search engine with user-defined possible modification(s) search criteria.

### Behavioral Analysis

A previous study showed that genetic reduction of STEP significantly reversed cognitive deficits in 6-mo-old 3xTg-AD mice [16]. Here we were interested in testing whether pharmacologic inhibition of STEP with TC-2153 had a similar beneficial effect in this AD mouse model. We also wanted to test whether TC-2153 had any effects on cognition in WT mice. Mice completed all tests in the following order: Y-maze alternation, NOR, and MWM. For all behavioral tests, WT or 3xTg-AD mice were randomly allocated to treatment with either vehicle or TC-2153.

### Open Field Activity

To assess locomotor activity and exploratory behavior, mice were placed in a square box (60×60×60 cm) and habituated for 5 min. Mice were treated with vehicle or TC-2153 (10 mg/kg, i.p.) 3 h prior to the exploration phase of the experiment. A video camera mounted directly above the box recorded the trials and ANY-maze software analyzed the distance traveled and time spent in the center of the box.

### Y-Maze Alternation Task

A crossover design was used in the Y-maze and NOR tasks, such that mice initially treated with vehicle (or TC-2153) were retested following a 15-d drug-free period and received TC-2153 (or vehicle). The Y-maze apparatus consisted of three dark gray arms (42×4.8×20 cm). Each mouse was treated with vehicle or TC-2153 (10 mg/kg, i.p.) 3 h prior to the experiment, after which they were placed at the end of one arm (the designated “start arm”) and allowed to freely explore the maze for 5 min. The total number of arm entries was recorded, as was the number of entries representing alternation behavior (i.e., sequential entry into all three arms). All four paws of the mouse had to enter an arm for it to count as an arm entry. Percentage spontaneous alternation  =  (number of alternations)/(total arm entries – 2). A crossover design was used after a drug-free period of 15 d, with groups previously treated with vehicle then receiving TC-2153 and vice versa. A total number of 20 WT and 11 AD mice were used in the Y-maze task.

### NOR Task

Mice were first habituated to the task by allowing them to explore an empty white open field box (60 cm×60 cm) for 5 min. Twenty-four hours later, mice were treated with vehicle or TC-2153 (10 mg/kg i.p.) 3 h prior to the sample phase. After the elapsed time, the mice completed the sample phase in which they were placed into the open field box with two identical objects located in the right and left corners. Mice were allowed to freely explore until they had accumulated a total of 30 s of object exploration (i.e., contact with the object with the nose and/or front paws), at which point the trial ended. The time spent with each object was recorded. Mice that were unable to complete the 30 s exploration within 20 min during the sample phase were excluded from the study (WT = 1 and AD = 3). Twenty-four hours later, mice completed the choice phase that was conducted in an identical manner to the sample phase except that one of the objects was substituted by a novel object and trial duration was set at 5 min. No drug treatments were given during the choice phase. Fifteen WT mice from the initial cohort were used to optimize the novel object conditions (to identify object pairings of inherent equal interest). Location of the novel object (left or right side) was counterbalanced to minimize possible bias. A crossover design was used, with a different set of objects after a 15-d drug-free period. DI was used to evaluate the effects of the TC-2153 compound on object memory in 6-mo-old 3xTg-AD mice. The DI was calculated for each subject by using the following formula: DI  =  (time spent exploring novel object – time spent exploring familiar object)/(total time spent exploring both objects). A DI of 0 is indicative of chance performance (i.e., no preference for one object compared to another), whereas a positive index (ranging from 0 to 1) indicates preference for novel object compared to familiar. In order to achieve greater statistical power, a second cohort of AD mice (*n* = 7) was run in the novel object test using a crossover design. Any value lower or higher than two times standard deviation away from the mean was considered an outlier and was excluded from the study (AD = 1). A total number of 9 WT and 16 AD mice were used in the NOR task.

### MWM

The reference memory version of the MWM task was performed as described previously [61]. A crossover design was not used in the MWM task, as the mice were randomly assigned to each treatment condition and can be exposed to the task only once. Briefly, animals were trained to swim in a 1.4 m diameter pool to find a submerged platform (14 cm in diameter) located 1 cm below the surface of water (24°C), rendered opaque by the addition of nontoxic white paint. Animals were pseudo-randomly started from a different position at each trial and used distal visual-spatial cues to find the hidden escape platform that remained in the center of the same quadrant throughout all training days. Training measures included escape latency to reach the platform, swim speed, and thigmotaxis. When animals failed to find the platform, they were guided to it and remained there for 10 s before removal. At 24 h after the acquisition phase, the platform was removed and a probe trial of 90 s was given to evaluate the number of entries in a circular zone (three times the platform diameter) positioned around the previous platform location (target zone) and in the opposite quadrants. To assess visual deficits and motivation to escape from water, the probe test was followed by a cued task (60 s; three trials per animal) during which the platform was visible. The visible platform was moved to different locations between each trial. After each trial, animals were immediately placed under a warming lamp to dry to prevent hypothermia. The experimenter was blind to mouse genotype when administering TC-2153 or vehicle to AD mice (AD-TC, *n* = 7; AD-Veh, *n* = 6) or WT mice (WT-TC, *n* = 13; WT-Veh, *n* = 12). Behavioral data from training, probe, and cued trials were acquired and analyzed using the ANY-maze automated tracking system (Stoelting, IL, USA).

### Data Analysis

A two-way analysis of variance (ANOVA) with genotype as the between-subject factor and treatment as the within subject factor was used for the Y-maze and object recognition tasks. Percent alternation (Y-maze) and DI (object recognition) were the dependent measures. Post hoc analyses were carried out using Bonferroni's multiple comparison tests as appropriate (GraphPad Prism, La Jolla, CA). In an older (12 mo) cohort of WT and 3xTg-AD mice, the exploration time (NOR task) did not meet the assumption of normality and equal variance, and raw data (seconds) were converted using square-root transformation followed by *t* test. For the MWM training and probe sessions, a three-way repeated measures ANOVA with two between-subject (Genotype, Treatment) and one within-subject (training day or quadrant) factor was used. Escape latency (training) and number of entries (probe) were the dependent measures (StatView, Cary, NC). Swim speed and escape latency during the probe and cued trials, respectively, were analyzed using a two-way ANOVA with genotype and treatment as the between-subject factors. Post hoc analyses were conducted on significant results. For cell-based assays, one-way ANOVA with post hoc Bonferroni test was used to determine statistical significance. All data were expressed as mean ± s.e.m.

## Supporting Information

Figure S1 Compounds were resynthesized and found to be inactive against STEP in the pNPP assay. Dose–response inhibition of STEP activity by commercial or resynthesized compounds was measured in the pNPP assay. Curves were obtained by fitting data to a second-order polynomial model.
(TIF)

Figure S2 Representative Western blots for histograms shown in Figure 2.
(TIF)

Figure S3 Representative Western blots for histograms shown in Figure 3.
(TIF)

Figure S4 TC-2153 stability in imidazole buffer. TC-2153 dissolved in water (A) and pH 7.0 imidazole buffer (B) were incubated for 1 h. For each experiment, the compound purity was determined using sensitive ^19^F-NMR, which is a sensitive technique for monitoring compound purity. As a control for monitoring modification of TC-2153, the compound was also incubated with 1 mM GSH in pH 7.0 imidazole buffer for 1 h (C), with compound modification clearly observed by ^19^F-NMR due to the appearance of multiple new peaks at a different chemical shift.
(TIF)

Figure S5 TC-2153 treatment does not generate ROS in cortical neuronal cultures (18 d *in vitro*). (A) H_2_O_2_ levels remain unchanged with 0.1, 1, or 10 µM TC-2153 treatment or with 200 U/ml superoxide dismutase (SOD) and catalase treatment. (B) ROS level, measured with the DCF fluorescence, is not increased with the indicated TC-2153 treatment.
(TIF)

Figure S6 MS/MS verification for the presence of a trisulfide peptide between C^465^ and C^472^ in TAT-STEP. The upper MS/MS spectrum shows the peaks observed for the fragmentation of the trisulfide peptide and assignments of the b- and y-ions. The inverted lower MS/MS spectrum shows the corresponding fragmentation of a peptide with a disulfide (from WT STEP in the absence of TC-2153), which has a mass difference of 32 Da (corresponding to a sulfur mass) from the trisulfide peptide. Peaks labeled in the lower spectrum with “*” are 32 Da less (corresponding to a Sulfur mass difference in the fragment ions) than their counterpart y-ions in the upper mass spectrum. The inset details the 32 Da mass differences for the y24^++^ fragment between the disulfide and trisulfide. Peaks labeled as “#” in the lower MS/MS spectrum does not have a mass shift between the modified and nonmodified peptide fragments because they do not contain the two cysteines that form the di- and tri-sulfide bridge.
(TIF)

Figure S7 No excessive and persistent thigmotaxic problem in 3xTg-AD mice in the MWM. There was no significant difference in percent time spent in zone A at the periphery of the tank as well as in zone B and C between 3xTg-AD and WT mice following treatment with vehicle or TC-2153 (three-way ANOVA).
(TIF)

Figure S8 TC-2153 has no effect on Aβ or phospho-tau levels in 12-mo-old 3xTg-AD mice. (A) Three hours prior to training, WT and 3xTg-AD mice were given vehicle or TC-2153 (10 mg/kg, i.p.). Time spent with either a novel or familiar object was recorded using ANY-maze software. Square-root transformation was used to meet the assumptions of normality and equal variance of the raw data. All histograms are presented as means ± s.e.m. Student's *t* test was applied to determine significance differences (*p<0.05; WT-Veh, *n* = 10; WT TC, *n* = 11; AD-Veh, *n* = 22; AD-TC, *n* = 19). (B) Cortical homogenates from vehicle or TC-2153–treated WT or 3xTg-AD mice were immunoprecipitated using 6E10 antibody and blotted with 6E10 antibody. CTFs and Aβ are indicated by arrowheads. Representative 7PA2-CM (Aβ-enriched conditioned medium) immunoprecipitation is shown on right panel. Data are presented as means ± s.e.m. Quantification of Aβ levels showed no significant difference in vehicle or TC-2153–treated 3xTg-AD brain samples (Student's *t* test, *p*>0.05; *n* = 3). (C) Cortical membrane fractions of vehicle or TC-2153–treated WT or 3xTg-AD mice were probed with p-tau (AT180) and total tau (HT7) antibody. Data are presented as means ± s.e.m. Quantification of p-tau levels showed no significant difference in vehicle or TC-2153–treated 3xTg-AD brain samples (Student's *t* test, *p*>0.05; *n* = 6).
(TIF)

Figure S9 Assay development. (A) Determination of K_m_ for pNPP with STEP. We reacted 200 nM STEP with different concentrations of pNPP. The OD_405_ was read at 5 min after the reaction was initiated. The K_m_ was determined to be 170 µM (*n* = 5). (B) Determination of K_m_ for DiFMUP with STEP. We reacted 200 nM STEP with different concentrations of DiFMUP. The fluorescence was read at 5 min after reactions started. K_m_ was determined to be 18.3 µM. The DiFMUP final concentration used in confirmatory screening was 20 µM. (C) Z' factor from representative plates from the primary screen for STEP inhibitors. The majority of the plates were between 0.7 and 0.9, indicating a robust assay.
(TIF)

Figure S10 Characterization of STEP. (A) pH dependency of STEP. Enzyme activity was assayed in buffers with varying pHs. (B) Salt dependency of STEP. Activity was assayed in the presence of increasing concentration of NaCl. (C) DMSO tolerance of STEP. STEP activity was determined in the presence of increasing concentrations of DMSO. (D) Stability of STEP at room temperature. STEP was left at room temperature for indicated time periods prior to initiation of the reaction by addition of pNPP substrate.
(TIF)

Figure S11 Synthesis of TC-2153. Scheme of large-scale synthesis of TC-2153.
(TIF)

Table S1 Eight compounds were selected for further characterization based on chemical structure and IC_50_ values.
(DOCX)

Table S2 *In vitro* inhibition of STEP by TC-2153. No change was observed *in vitro* in the activity of STEP or inhibition by TC-2153 when changing the buffer from pH 7.0 to pH 6.0. As it is physiologically relevant, further studies were conducted at pH 7.0. The addition of the antioxidant enzymes catalase and superoxide dismutase had no effect on the inhibition of STEP by TC-2153. Reduced glutathione decreased the inhibitory activity of TC-2153 by two orders of magnitude.
(DOCX)

Table S3 Primary and secondary antibodies used in this study.
(DOCX)

Text S1 Supplemental methods and results.
(DOC)

## Abbreviations

3xTg-AD, triple transgenic Alzheimer mice; Aβ, beta-amyloid; AD, Alzheimer's disease; AMPAR, α-amino-3-hydroxy-5-methyl-4-isoxazolepropionic acid receptor; DCF, dichlorofluorescein diacetate; DiFMUP, 6,8-difluoro-4-methylumbelliferyl phosphate; HPLC, high-performance liquid chromatography; IC_50_, median inhibition concentration; i.p., intraperitoneal; GSH, reduced glutathione; KO, knock out; MAPK, mitogen-activated protein kinase; MWM, Morris water maze; NMDAR, N-methyl-D-aspartate receptor; NOR, novel object recognition; PAGE, polyacrylamide electrophoresis; pNPP, *para*-nitrophenyl phosphate; ppm, parts per million; PTP, protein tyrosine phosphatase; ROS, reactive oxygen species; SDS, sodium dodecyl sulfate; SOD, superoxide dismutase; STEP, STriatal-Enriched protein tyrosine Phosphatase; TC-2153, benzopentathiepin 8-(trifluoromethyl)-1,2,3,4,5-benzopentathiepin-6-amine hydrochloride; Tg-2576, transgenic mouse line that contains mutations in the amyloid precursor protein; WT, wild type
