# Supplementary figures and images for: Inhibitor of the Tyrosine Phosphatase STEP Reverses Cognitive Deficits in a Mouse Model of Alzheimer's Disease
Source: PLoS Biol. 2014 Aug 5;12(8):e1001923. doi: 10.1371/journal.pbio.1001923 (PMC4122355; doi:10.1371/journal.pbio.1001923)

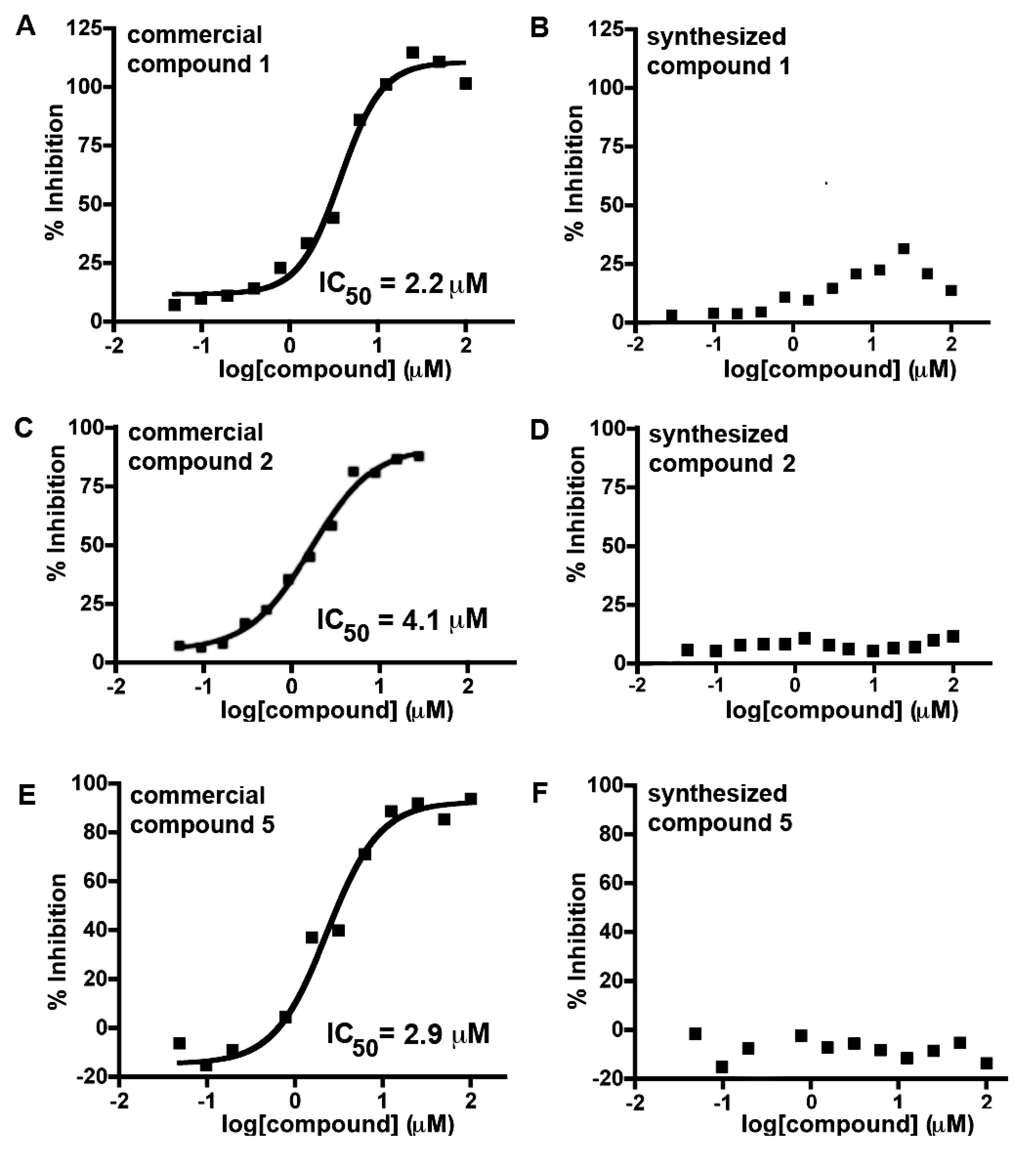

Supplement: Figure S1 — Compounds were resynthesized and found to be inactive against STEP in the pNPP assay. Dose–response inhibition of STEP activity by commercial or resynthesized compounds was measured in the pNPP assay. Curves were obtained by fitting data to a second-order polynomial model. (TIF) [file pbio.1001923.s001.tif]

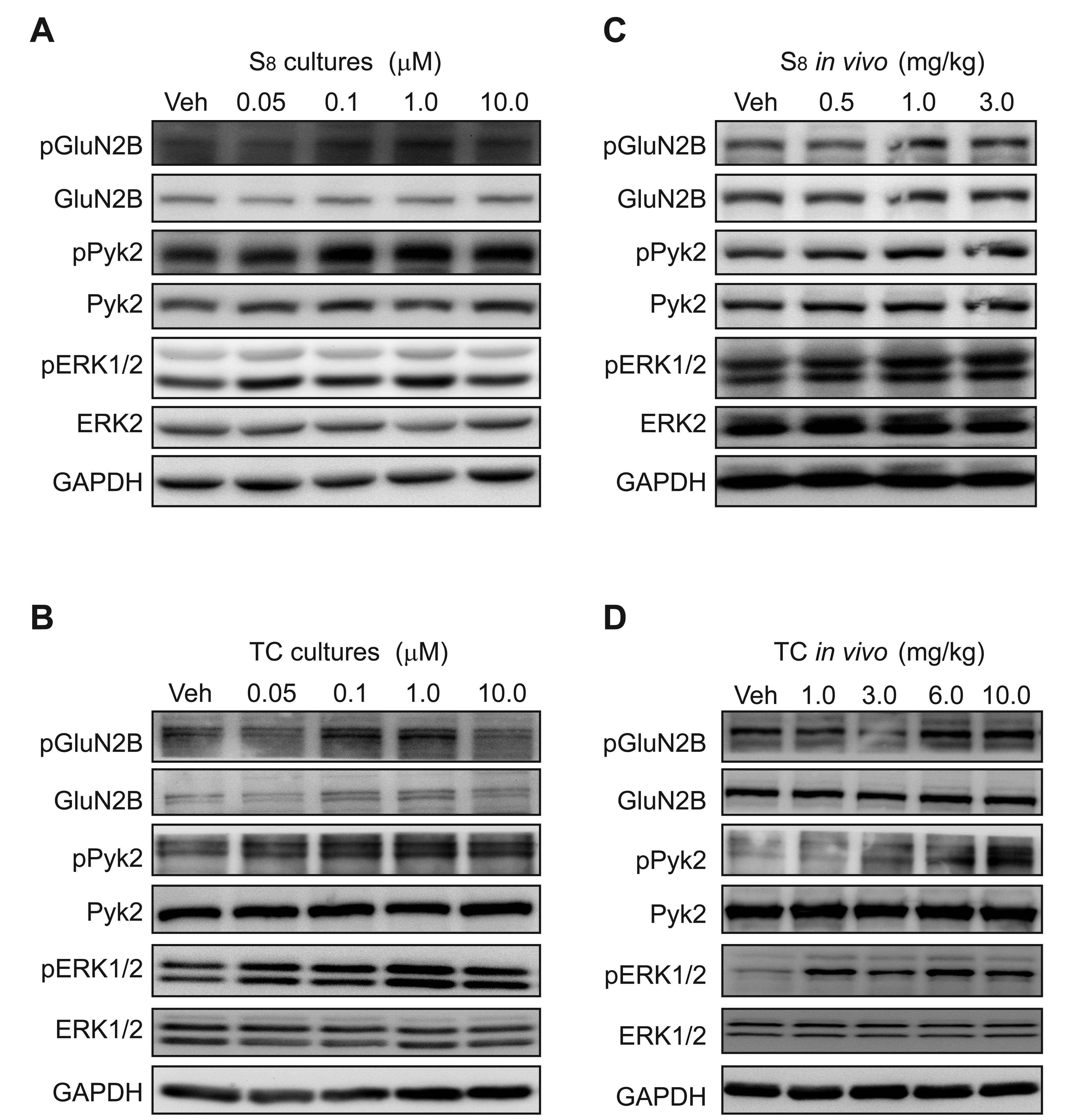

Supplement: Figure S2 — Representative Western blots for histograms shown in Figure 2. (TIF) [file pbio.1001923.s002.tif]

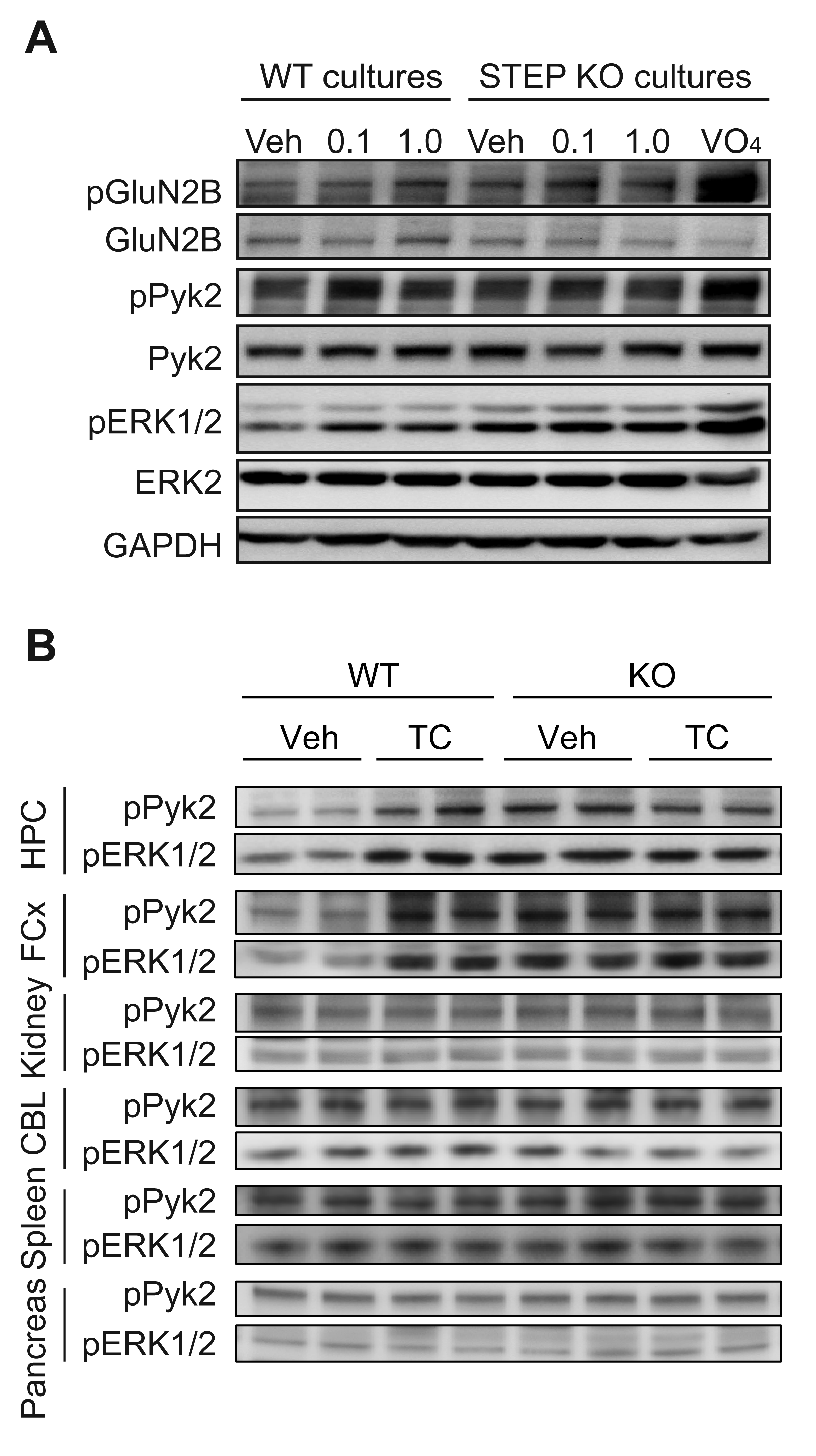

Supplement: Figure S3 — Representative Western blots for histograms shown in Figure 3. (TIF) [file pbio.1001923.s003.tif]

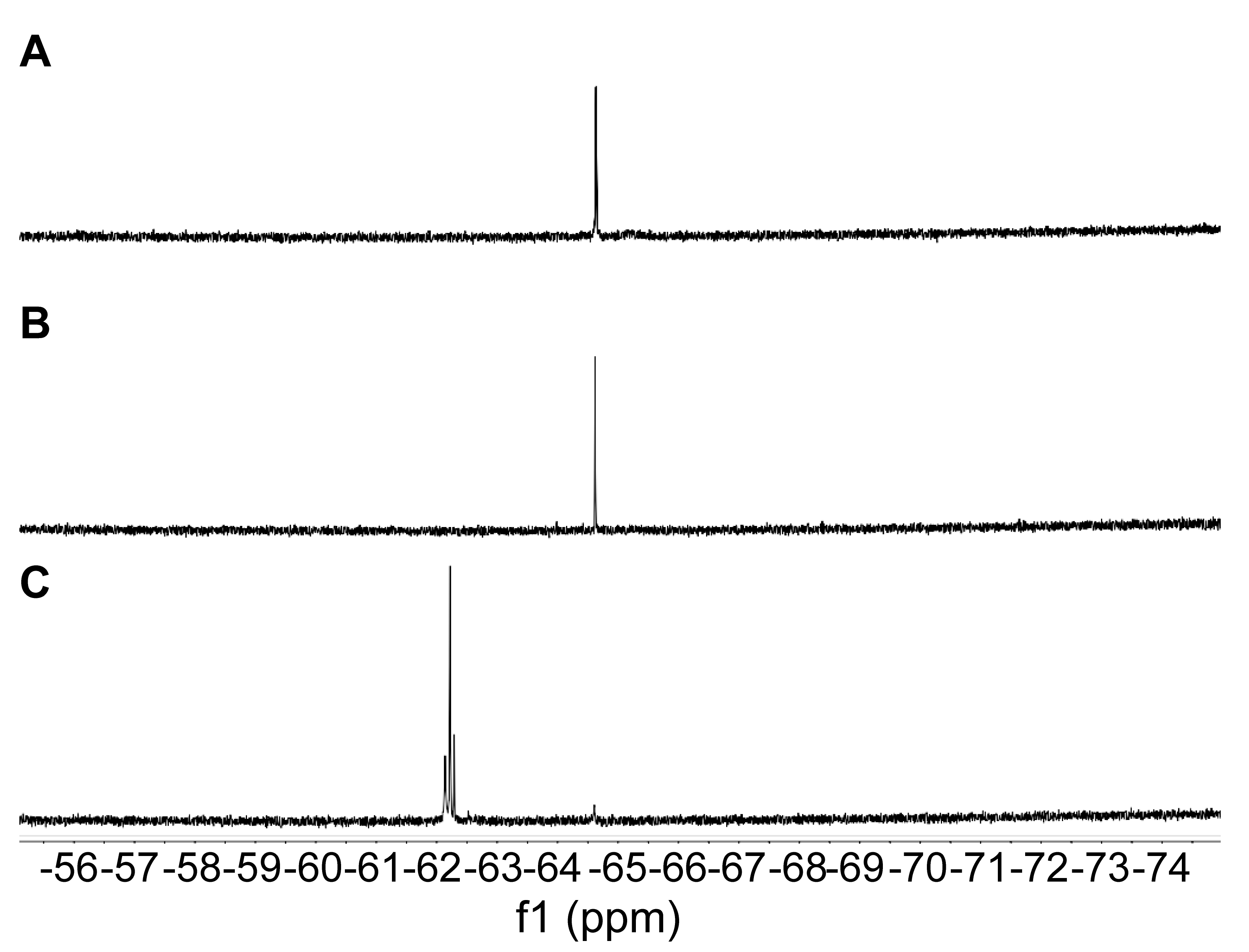

Supplement: Figure S4 — TC-2153 stability in imidazole buffer. TC-2153 dissolved in water (A) and pH 7.0 imidazole buffer (B) were incubated for 1 h. For each experiment, the compound purity was determined using sensitive 19F-NMR, which is a sensitive technique for monitoring compound purity. As a control for monitoring modification of TC-2153, the compound was also incubated with 1 mM GSH in pH 7.0 imidazole buffer for 1 h (C), with compound modification clearly observed by 19F-NMR due to the appearance of multiple new peaks at a different chemical shift. (TIF) [file pbio.1001923.s004.tif]

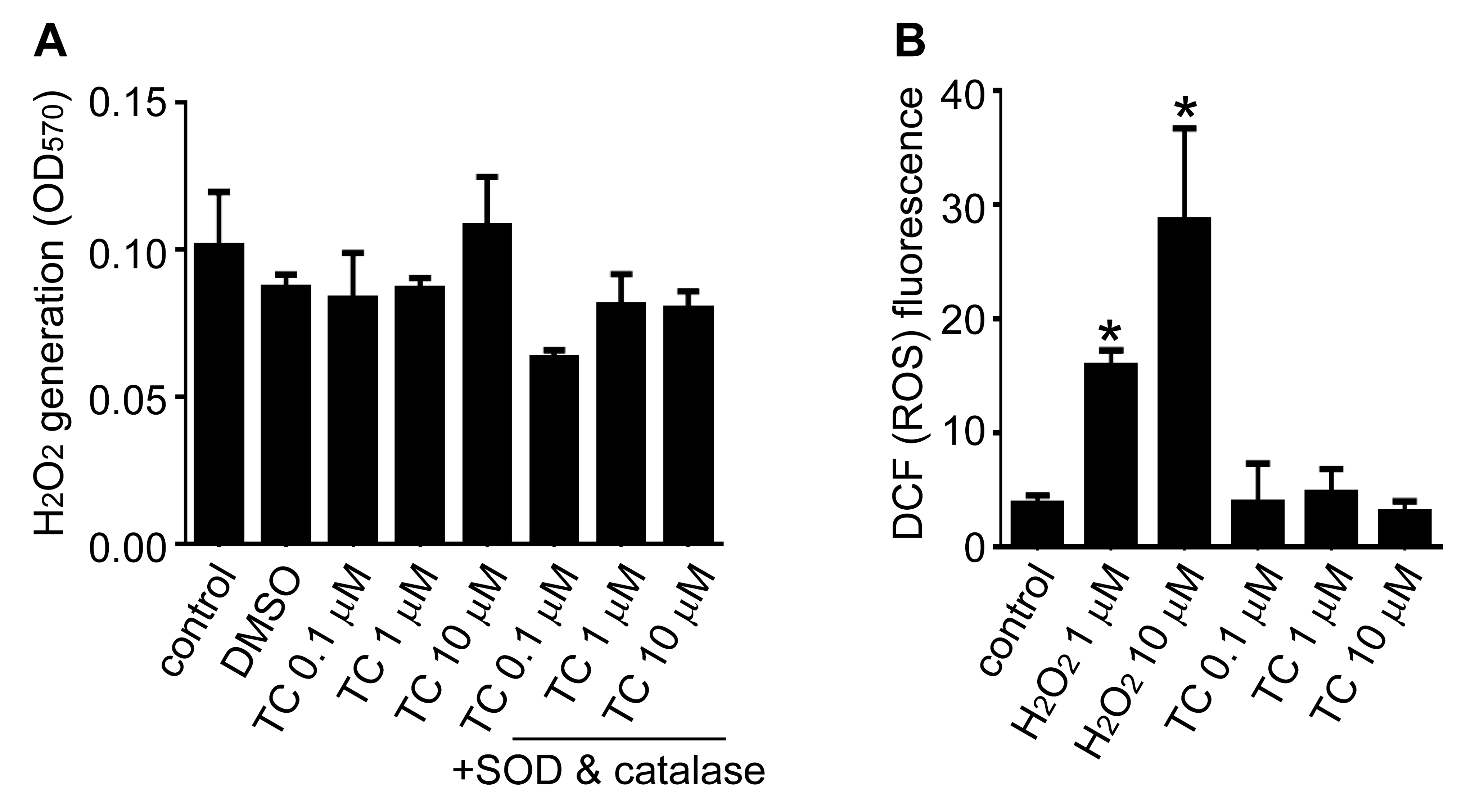

Supplement: Figure S5 — TC-2153 treatment does not generate ROS in cortical neuronal cultures (18 d in vitro). (A) H2O2 levels remain unchanged with 0.1, 1, or 10 µM TC-2153 treatment or with 200 U/ml superoxide dismutase (SOD) and catalase treatment. (B) ROS level, measured with the DCF fluorescence, is not increased with the indicated TC-2153 treatment. (TIF) [file pbio.1001923.s005.tif]

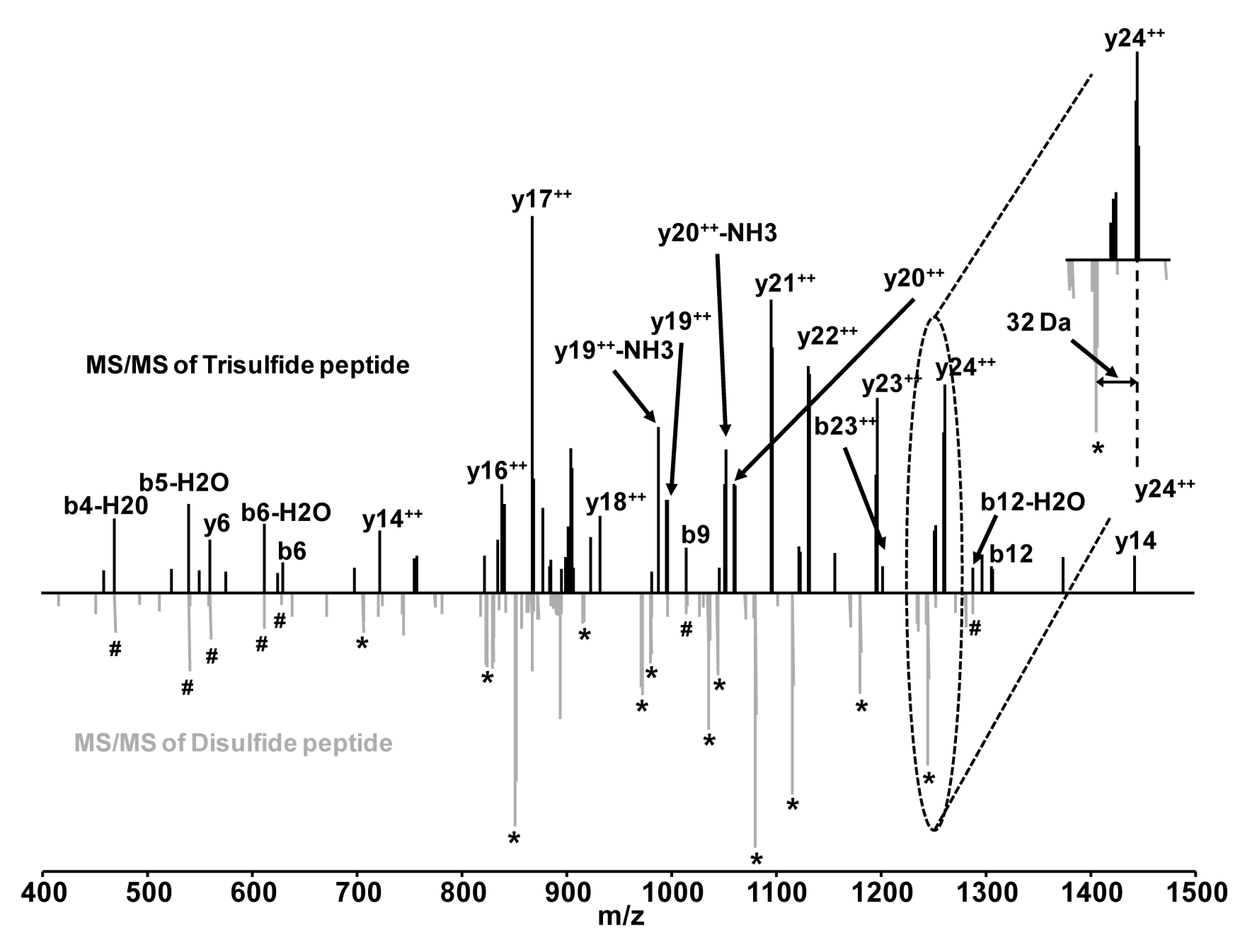

Supplement: Figure S6 — MS/MS verification for the presence of a trisulfide peptide between C465 and C472 in TAT-STEP. The upper MS/MS spectrum shows the peaks observed for the fragmentation of the trisulfide peptide and assignments of the b- and y-ions. The inverted lower MS/MS spectrum shows the corresponding fragmentation of a peptide with a disulfide (from WT STEP in the absence of TC-2153), which has a mass difference of 32 Da (corresponding to a sulfur mass) from the trisulfide peptide. Peaks labeled in the lower spectrum with “*” are 32 Da less (corresponding to a Sulfur mass difference in the fragment ions) than their counterpart y-ions in the upper mass spectrum. The inset details the 32 Da mass differences for the y24++ fragment between the disulfide and trisulfide. Peaks labeled as “#” in the lower MS/MS spectrum does not have a mass shift between the modified and nonmodified peptide fragments because they do not contain the two cysteines that form the di- and tri-sulfide bridge. (TIF) [file pbio.1001923.s006.tif]

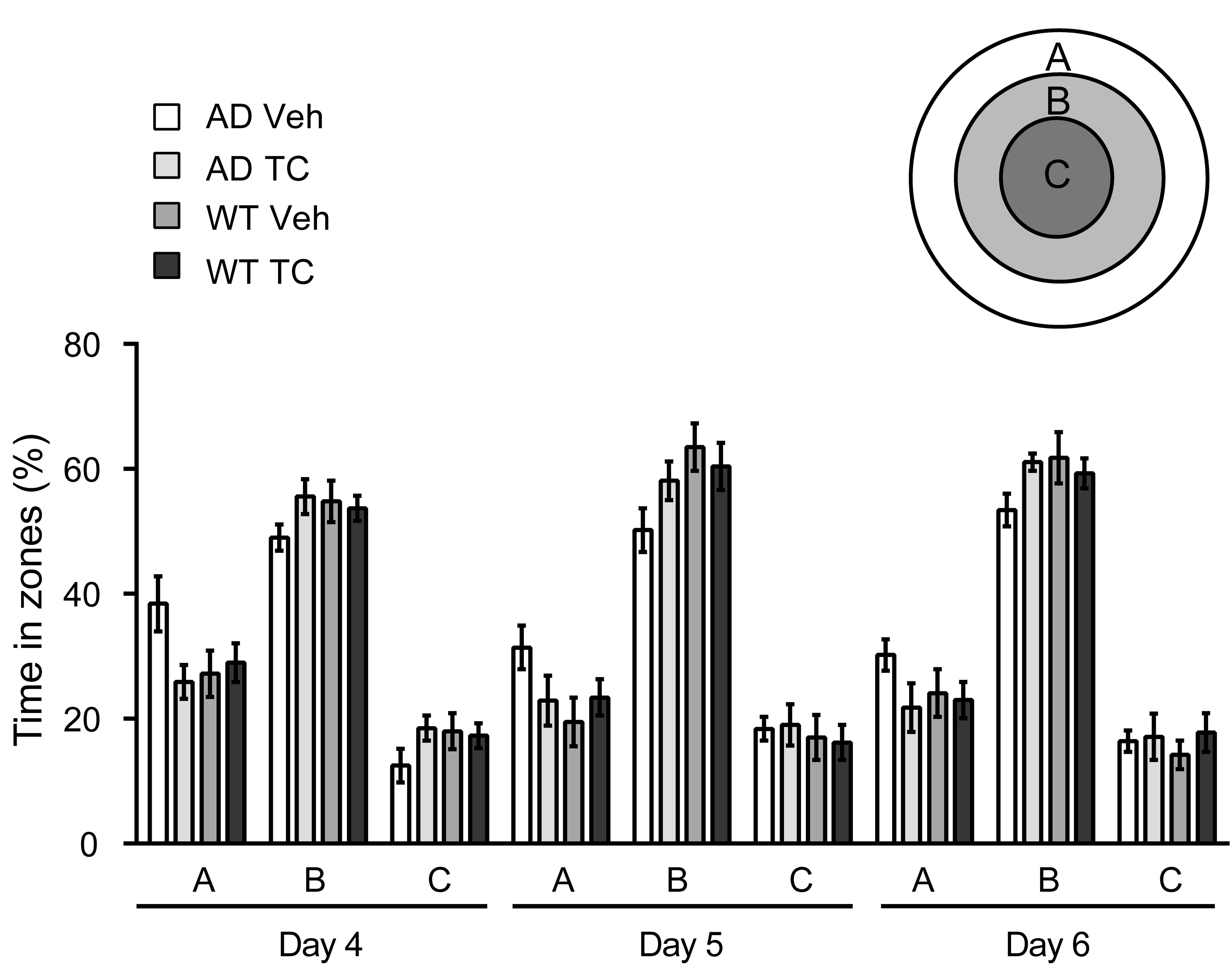

Supplement: Figure S7 — No excessive and persistent thigmotaxic problem in 3xTg-AD mice in the MWM. There was no significant difference in percent time spent in zone A at the periphery of the tank as well as in zone B and C between 3xTg-AD and WT mice following treatment with vehicle or TC-2153 (three-way ANOVA). (TIF) [file pbio.1001923.s007.tif]

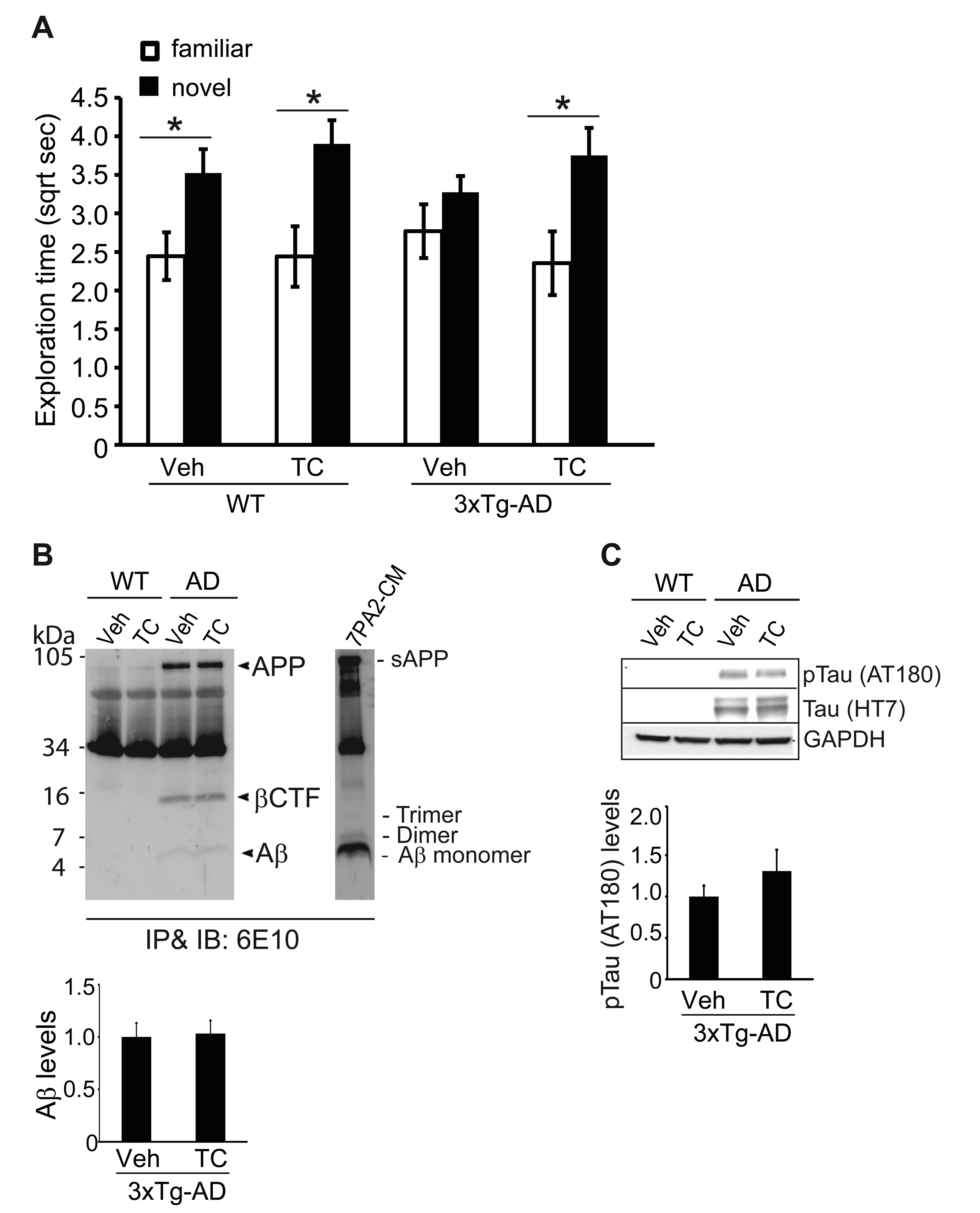

Supplement: Figure S8 — TC-2153 has no effect on Aβ or phospho-tau levels in 12-mo-old 3xTg-AD mice. (A) Three hours prior to training, WT and 3xTg-AD mice were given vehicle or TC-2153 (10 mg/kg, i.p.). Time spent with either a novel or familiar object was recorded using ANY-maze software. Square-root transformation was used to meet the assumptions of normality and equal variance of the raw data. All histograms are presented as means ± s.e.m. Student's t test was applied to determine significance differences (*p<0.05; WT-Veh, n = 10; WT TC, n = 11; AD-Veh, n = 22; AD-TC, n = 19). (B) Cortical homogenates from vehicle or TC-2153–treated WT or 3xTg-AD mice were immunoprecipitated using 6E10 antibody and blotted with 6E10 antibody. CTFs and Aβ are indicated by arrowheads. Representative 7PA2-CM (Aβ-enriched conditioned medium) immunoprecipitation is shown on right panel. Data are presented as means ± s.e.m. Quantification of Aβ levels showed no significant difference in vehicle or TC-2153–treated 3xTg-AD brain samples (Student's t test, p>0.05; n = 3). (C) Cortical membrane fractions of vehicle or TC-2153–treated WT or 3xTg-AD mice were probed with p-tau (AT180) and total tau (HT7) antibody. Data are presented as means ± s.e.m. Quantification of p-tau levels showed no significant difference in vehicle or TC-2153–treated 3xTg-AD brain samples (Student's t test, p>0.05; n = 6). (TIF) [file pbio.1001923.s008.tif]

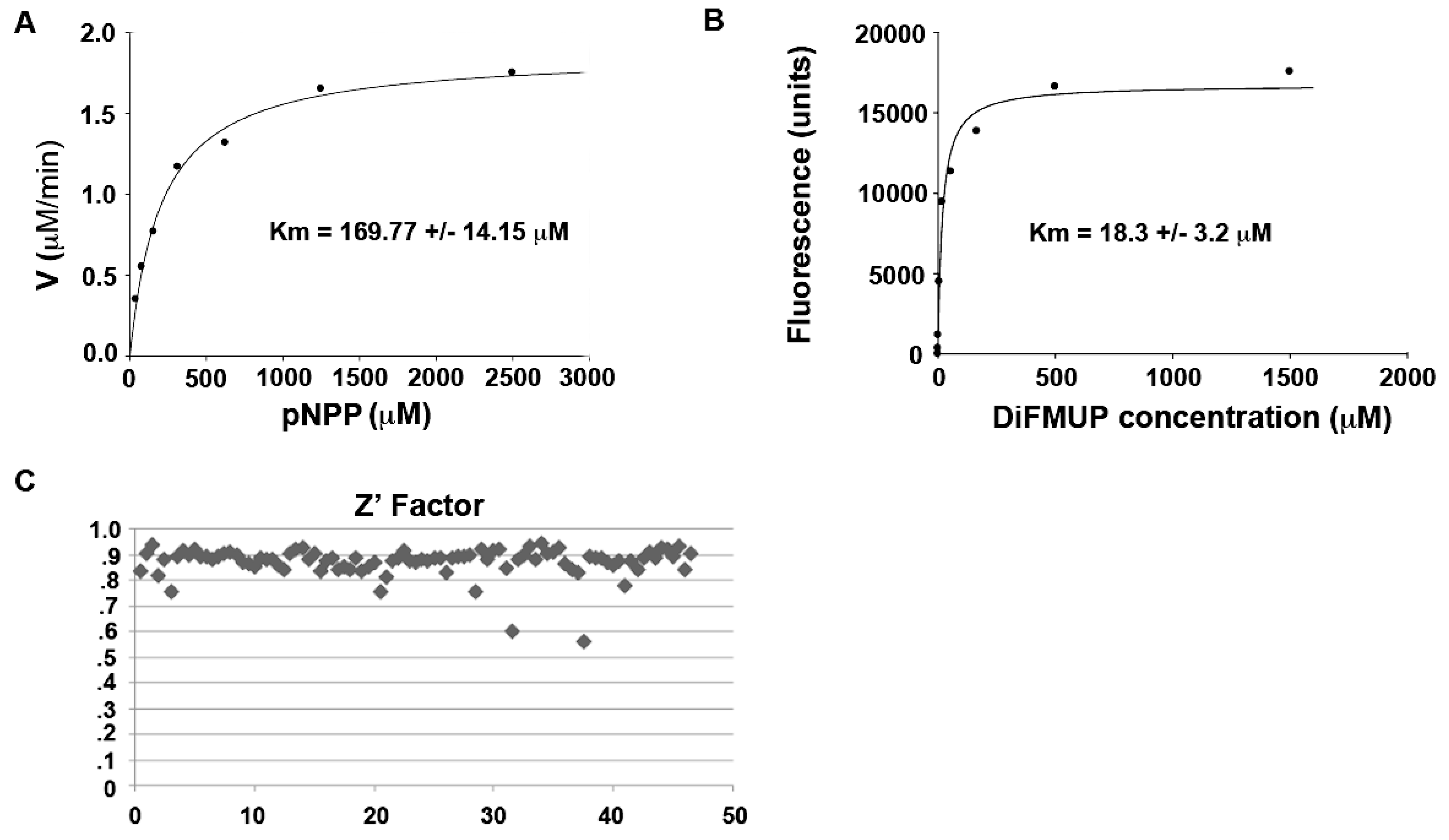

Supplement: Figure S9 — Assay development. (A) Determination of Km for pNPP with STEP. We reacted 200 nM STEP with different concentrations of pNPP. The OD405 was read at 5 min after the reaction was initiated. The Km was determined to be 170 µM (n = 5). (B) Determination of Km for DiFMUP with STEP. We reacted 200 nM STEP with different concentrations of DiFMUP. The fluorescence was read at 5 min after reactions started. Km was determined to be 18.3 µM. The DiFMUP final concentration used in confirmatory screening was 20 µM. (C) Z' factor from representative plates from the primary screen for STEP inhibitors. The majority of the plates were between 0.7 and 0.9, indicating a robust assay. (TIF) [file pbio.1001923.s009.tif]

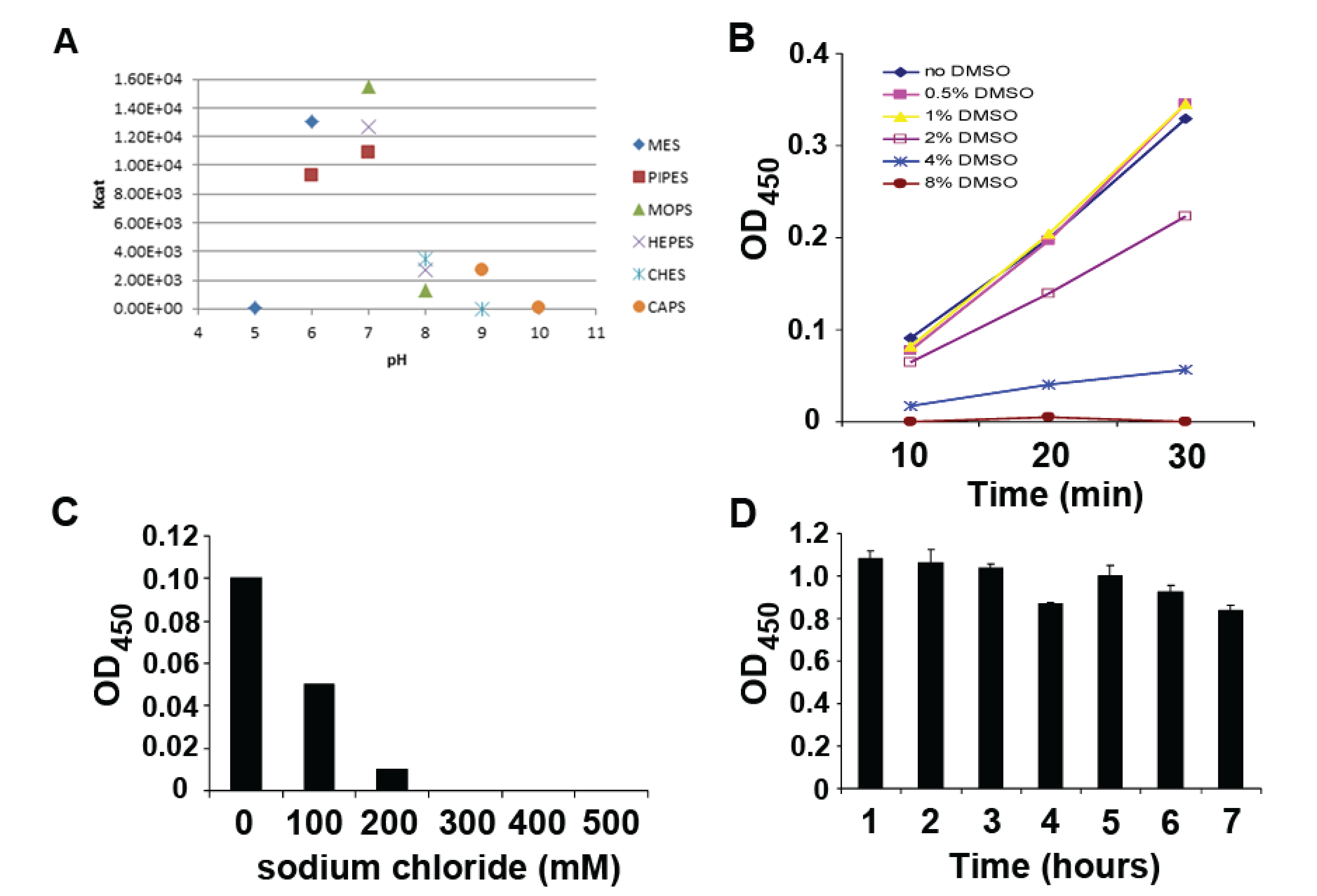

Supplement: Figure S10 — Characterization of STEP. (A) pH dependency of STEP. Enzyme activity was assayed in buffers with varying pHs. (B) Salt dependency of STEP. Activity was assayed in the presence of increasing concentration of NaCl. (C) DMSO tolerance of STEP. STEP activity was determined in the presence of increasing concentrations of DMSO. (D) Stability of STEP at room temperature. STEP was left at room temperature for indicated time periods prior to initiation of the reaction by addition of pNPP substrate. (TIF) [file pbio.1001923.s010.tif]

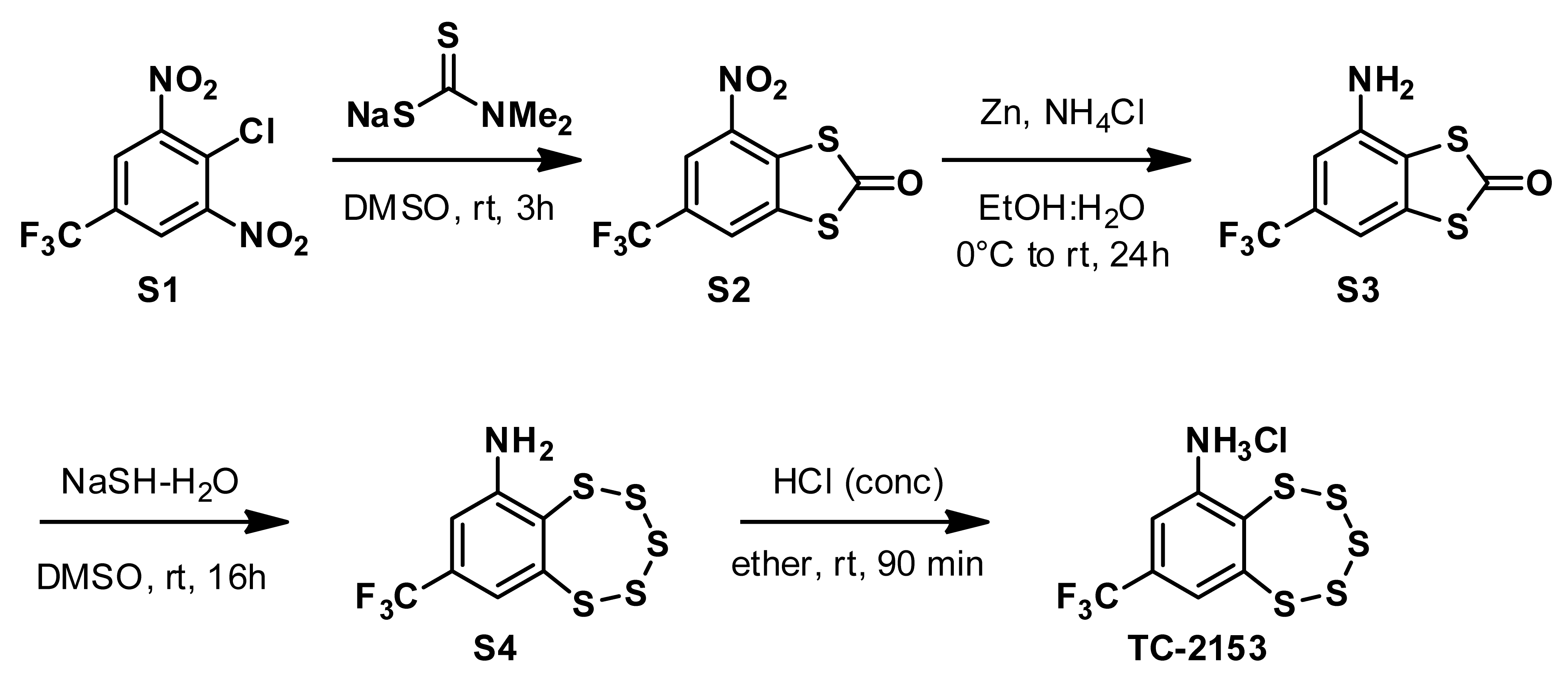

Supplement: Figure S11 — Synthesis of TC-2153. Scheme of large-scale synthesis of TC-2153. (TIF) [file pbio.1001923.s011.tif]
