# Supplementary material for: Inhibitor of the Tyrosine Phosphatase STEP Reverses Cognitive Deficits in a Mouse Model of Alzheimer's Disease
Source: PLoS Biol. 2014 Aug 5;12(8):e1001923. doi: 10.1371/journal.pbio.1001923 (PMC4122355; doi:10.1371/journal.pbio.1001923)
Supplement: Table S1 — Eight compounds were selected for further characterization based on chemical structure and IC50 values. (DOCX) [file pbio.1001923.s012.docx]

**Table S1.** **Eight compounds were selected for further characterization based on chemical structure and IC_50_ values.**

| **Compound** | **LDN ID #** | **Structure** | **IC_50_ (μM)** |
| --- | --- | --- | --- |
| 1 | 33960 | 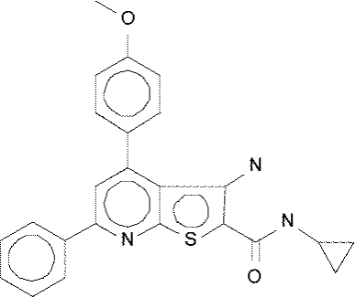 | 2.2 |
| 2 | 211885 | **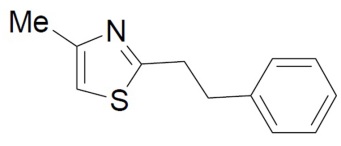** | 4.1 |
| 3 | 3358 | 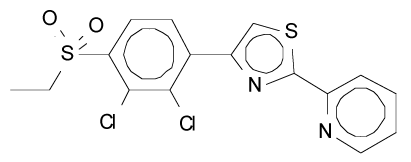 | 9.7 |
| 4 | 1485 | 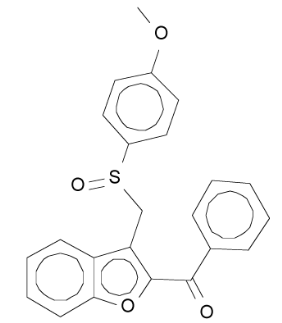 | 8.7 |
| 5 | 88594 | 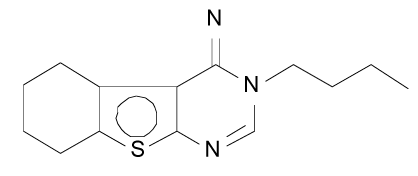 | 2.9 |
| 6 | 99744 | 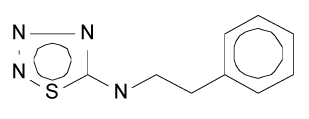 | 1.4 |
| 7 | 46512 | 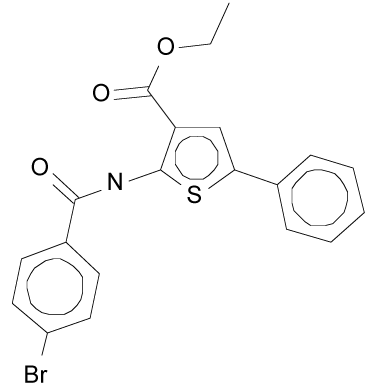 | 4.9 |
| 8 | 72221 | 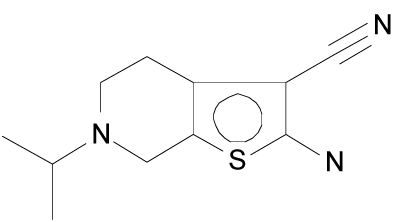 | 0.98 |
