# Supplementary material for: Inhibitor of the Tyrosine Phosphatase STEP Reverses Cognitive Deficits in a Mouse Model of Alzheimer's Disease
Source: PLoS Biol. 2014 Aug 5;12(8):e1001923. doi: 10.1371/journal.pbio.1001923 (PMC4122355; doi:10.1371/journal.pbio.1001923)
Supplement: Table S2 — In vitro inhibition of STEP by TC-2153. No change was observed in vitro in the activity of STEP or inhibition by TC-2153 when changing the buffer from pH 7.0 to pH 6.0. As it is physiologically relevant, further studies were conducted at pH 7.0. The addition of the antioxidant enzymes catalase and superoxide dismutase had no effect on the inhibition of STEP by TC-2153. Reduced glutathione decreased the inhibitory activity of TC-2153 by two orders of magnitude. (DOCX) [file pbio.1001923.s013.docx]

**Table S2. *In vitro* inhibition of STEP by TC-2153.**

| Conditions | IC_50_ (nM) |
| --- | --- |
| pH 7.0*^a^* | 24.6 ± 0.8 |
| pH 6.0*^b^* | 23.8 ± 1.6 |
| pH 7.0 + catalase*^b,c^* | 26.2 ± 0.6 |
| pH 7.0 + SOD*^b,d^* | 18.6 ± 0.8 |
| pH 7.0 + catalase + SOD*^b,c,d^* | 24.5 ± 4.2 |
| pH 7.0 + GSH*^b,e^* | 8,790 ± 430 |

*^a^*Mean ± s.e.m. (n = 4). *^b^*Mean ± s.e.m. (n = 2). *^c^*80 U/mL catalase. *^d^*100 U/mL superoxide dismutase (SOD). *^e^*1 mM reduced glutathione (GSH).
