# Supplementary material for: Inhibitor of the Tyrosine Phosphatase STEP Reverses Cognitive Deficits in a Mouse Model of Alzheimer's Disease
Source: PLoS Biol. 2014 Aug 5;12(8):e1001923. doi: 10.1371/journal.pbio.1001923 (PMC4122355; doi:10.1371/journal.pbio.1001923)
Supplement: Table S3 — Primary and secondary antibodies used in this study. (DOCX) [file pbio.1001923.s014.docx]

**Table S3. Primary and secondary antibodies used in this study.**

| Antibody | Format | Immunogen | Host | Dilution | Source |
| --- | --- | --- | --- | --- | --- |
| Anti-pTyr^1472^ GluN2B | Whole IgG, unconjugated | Synthetic phosphopeptide | Rabbit | 1:1000 | Cell Signaling Technology, Danvers, MA |
| Anti-GluN2B | Whole IgG, unconjugated | C-terminus of mouse GluN2B | Rabbit | 1:1000 | Millipore |
| Anti-pTyr^402^ Pyk2 | Whole IgG, unconjugated | Synthetic phosphopeptide of human Pyk2 | Rabbit | 1:1000 | Invitrogen |
| Anti-Pyk2 | IgG2a | C-terminus of human Pyk2 | Mouse | 1:1000 | Cell Signaling Technology |
| Anti-pTyr^204^ ERK1/2 | Whole IgG, unconjugated | Synthetic phosphopeptide | Mouse | 1:500 | Santa Cruz Biotechnology |
| Anti-ERK2 | Whole IgG, unconjugated | C-terminus of rat p44 MAP Kinase | Rabbit | 1:20,000 | Cell Signaling Technology |
| Anti-GAPDH, clone 6c5 | IgG1, unconjugated | Purified protein from rabbit muscle | Mouse | 1:20,000 | Millipore |
| Anti-rabbit | Whole IgG peroxidase-conjugated | Rabbit Fc | Donkey | 1:10,000 | Amersham Biosciences |
| Anti-mouse | Whole IgG peroxidase-conjugated | Mouse Fc | Sheep | 1:5,000 | Amersham Biosciences |
