# Supplementary material for: Inhibitor of the Tyrosine Phosphatase STEP Reverses Cognitive Deficits in a Mouse Model of Alzheimer's Disease
Source: PLoS Biol. 2014 Aug 5;12(8):e1001923. doi: 10.1371/journal.pbio.1001923 (PMC4122355; doi:10.1371/journal.pbio.1001923)
Supplement: Text S1 — Supplemental methods and results. (DOC) [file pbio.1001923.s015.doc]

**Material and Methods**

**Assay development**

The specific activity of STEP was determined based on the dephosphorylation of a generic phosphatase substrate, pNPP, the Michaelis-Menten constant (Km) of which was determined to be 170 μM for STEP (Figure S9A). The activity of the enzyme was measured based on the detection of the dephosphorylated product, *p*-nitrophenol, by measuring its absorbance at OD405 nm. Because many small molecules show absorbance at similar wavelengths, potentially masking compound activity, an alternate assay was employed for HTS. We used the molybdate-malachite green-based reagent, which forms a green colored complex with the released free phosphate from the reaction, and measured absorbance of the complex at OD632 nm. Both standard curve and kinetic constants of the malachite green assay were confirmed.

A secondary, orthogonal assay was developed for additional confirmation using the fluorescence-based substrate DiFMUP. TheKm for DiFMUP with STEP was determined to be 18.3 μM(Figure S9B). The 121 hits were tested in a dose-response DiFMUP assay, and 91 compounds confirmed in the secondary assay. Z’ factors for all screens ranged between 0.7 - 0.9, and any plate with Z’ < 0.5 was repeated (Figure S9C).

We optimized assay conditions by testing various buffer conditions, DMSO tolerance, salt concentrations, and stability at room temperature (Figure S10). Optimization of the buffer and pH was performed with buffers with pH ranging from 5.0 to 10.0. Rate constant (kcat) values were obtained for each buffer and pH point, and STEP showed good catalytic capacity at pH 6 in 50 mM MES buffer, which was used for the HTS. Salt dependency of STEP was tested in pNPP reactions with different concentrations of NaCl, ranging from 100 to 500 mM in MES buffer. The results show that STEP activity decreased by 50% in the presence of 100 mM sodium chloride and was dramatically reduced in the presence of 300 mM or higher concentrations. Thus, no salt was added to the MES buffer. Due to the fact that the compounds in the library are dissolved in DMSO, we tested the DMSO tolerance of the enzyme. Up to 1% DMSO did not affect STEP activity. Stability of the STEP enzyme at room temperature was also tested. The results showed that STEP is stable at room temperature for up to 70 min, sufficient time period to complete the screening assay.

**Compound library**

The compound library consisted of ~150,000 small molecules, including compounds approved by the Food and Drug Administration (FDA), a purified natural products library, compounds purchased from Peakdale (High Peak, UK), Maybridge Plc. (Cornwall, UK), Cerep (Paris, France), Bionet Research Ltd. (Cornwall, UK), Prestwick (Ilkirch, France), Specs and Biospecs (CP Rijswijk, the Netherlands), ENAMINE (Kiev, Ukraine), I.F. Lab LTD (Burlington, Canada), and Chemical Diversity Labs (San Diego, CA), and small molecules from different academic institutions. Compounds were selected from different vendors by applying a series of filters, including examination of clogP and predicted solubility. All of the small molecules generally adhere to Lipinski’s rules (i.e. molecular weight < 500, H-bond donors < 5, H-bond acceptors < 10, and logP < 5) and contain a low proportion of known toxicophores (i.e. Michael acceptors and alkylating agents) and unwanted functionalities (i.e. imines, thiols, and quarternary amines) and have been optimized to maximize molecular diversity. Compound source plates for the assay were prepared by spotting 0.4 μL of 1.67 mM compound in DMSO in each well of a Greiner 384-well plate, with columns 23 and 24 spotted with neat DMSO for positive and negative controls. These plates were then sealed with aluminum plate seals and stored at -20°C. Phosphatase reactions using the generic phosphatase substrate pNPP were performed in MES buffer.

**High-throughput screening (HTS)**

HTS was performed using pNPP assay running at a Km concentration for pNPP (250 μM). After adding 10 μL MES buffer (50 mM, pH 6) to each well, compounds (11 μM final concentration) and STEP (30 μL, 100 nM final concentration) were preincubated for 30 min at room temperature (void light). The reaction was started by adding pNPP (20 μL; 250 μM final concentration), and after 30 min the reaction was stopped by adding 15 μL of malachite green reagent. Absorbance of the reaction mixture was read at OD632 nm**.** We determined % inhibition using the following equation: % Inhibition = 100 × [1-(sample – low control) / (high control - low control)].Approximately 1,120 initial hits were tested in an orthogonal assay using a 6,8-difluoro-4-methylumbelliferyl phosphate (DiFMUP) as substrate. STEP (30 μL of 100 nM) was preincubated with compounds for 30 min at room temperature (void light), DiFMUP (30 μL, 20 μM final concentration) was added, and the reaction was run for 20 min before stopping with sodium orthovanadate (5 μL of 1 mM). The fluorescence emission of the reaction mixture was read using an excitation wavelength of OD355 nm, and reading the emission at OD460 nm.Compounds exhibiting ≥ 97% inhibition were selected for validation in five-point dose-response assays (0.41, 1.22, 3.67, 11, 33 μM) with duplicates, using both pNPP and DiFMUP.

**Synthetic procedures for the preparation of TC-2153 on a large scale**

**General Methods**

Unless otherwise noted, all reagents and solvents were obtained from commercial suppliers and used without further purification. Diethyl ether and CH2Cl2 were passed through a column of activated alumina (type A2, 12 × 32, Purify Co.) under nitrogen pressure immediately prior to use. All 1H, 19F and 13C NMR spectra were obtained at room temperature on a Bruker AVB-400 or AVB-500 spectrometer. NMR chemical shifts are reported in ppm relative to CHCl3 (7.26), or DMSO (2.50) for 1H, trifluoroacetic acid (−76.55) for 19F, and CHCl3 (77.16) or DMSO (39.52) for 13C. Mass spectrometry (HRMS, ESI, GCMS) are reported in m/z. Chromatography was performed with SiliCycle SiliaFlash® P60 230–400 mesh silica gel. Melting points were recorded on an Electrothermal Melting Point Apparatus and are uncorrected.

**4-Nitro-6-(trifluoromethyl)benzo-1,3-dithio-2-one (S2).** Modifying a known literature procedure , a 1 L round bottomed flask was charged with a magnetic stir bar, 135 g (0.500 mol) of 4-chloro-3,5-dinitrobenzotrifluoride (**S1**) and 70 mL of DMSO. The reaction flask was suspended in an ambient temperature water bath. A solution of sodium dimethyldithiocarbamate dihydrate (89.7 g, 0.500 mol) in DMSO (200 mL) was added dropwise *via* cannula over 2 h. After addition, the reaction mixtures was stirred for 1 h in the water bath and was quenched by addition of 400 mL of water. The reaction mixture was transferred to a larger flask and was diluted to 2 L with water, then was extracted with CH2Cl2 (3 × 1 L and then 3 ×500 mL). The combined organic layer was condensed to approximately 2 L, washed with brine (1 × 1 L), and dried by stirring over MgSO4 for 20 min. The remaining solvent was removed on a rotary evaporator. The resulting solid was purified by SiO2 flash chromatography using 4 L of SiO2 and was dry-loaded onto the column with approximately 200 mL of SiO2. The product was eluted first with 9:1 and then 4:1 hexanes:EtOAc to yield the product as a light orange solid (61.4 g, 43.6 %). The NMR data correlates with the published data .

**4-Amino-6-(trifluoromethyl)benzo-1,3-dithio-2-one (S3).** The procedure is based on an existing literature procedure with some modifications . A 2 L round bottomed three-neck Morten flask was charged with 300 mL of water, 300 mL of EtOH, 214 g of zinc metal (3.27 mol, 15 equiv), 93.3 g (1.74 mol, 8 equiv) of ammonium chloride and was equipped with a mechanical stirrer. The reaction flask was placed into an ice water bath. Nitro compound **S2** (61.3 g, 0.218 mol) was added as a solid portion-wise with mechanical stirring over 15 min. The ice bath was removed, and the reaction solution was allowed to warm to room temperature with mechanically stirring for 24 h. The reaction mixture was neutralized with the addition of 100 mL of saturated aqueous sodium bicarbonate and was filtered through a fritted funnel to remove solids. The filter cake was washed with water (500 mL) and CH2Cl2 (1 L). The layers were separated and the water layer was extracted with CH2Cl2 (3 ×500 mL). The combined organic layers were condensed to 1 L, washed with brine (1 L), and dried by stirring over MgSO4 for 20 min. The solvent was removed on a rotary evaporator, yielding 45.8 g (83.5 %) of the title compound as an off-white solid, m.p. 148-151 °C. 1H NMR (500 MHz, CDCl3) δ 7.19 (d, J = 1.5 Hz, 1H), 6.88 (d, J = 1.5 Hz, 1H), 3.98 (s, 2H). 19F NMR (376 MHz, CDCl3) δ -62.85. 13C NMR (126 MHz, CDCl3) δ 188.20, 141.63, 134.16, 130.61 (q, J = 33.0 Hz), 123.90 (q, J = 272.7 Hz), 121.05, 110.28 (q, J = 4.1 Hz), 109.84 (q, J = 3.7 Hz). HRMS-ESI (*m/z*): calcd for C8H5F3NOS2+ [M+H]+ 251.9759; found 251.9163.

**8-(Trifluoromethyl)-1,2,3,4,5-benzopentathiepin-6-amine (S4).** This reaction was performed on 15 g batches to maintain a reasonable solvent volume during the extraction steps (vide infra). A 2 L round bottomed three-neck Morten flask was charged with 22.3 g of NaSH-H2O (300 mmol, 0.5 M final concentration), 500 mL of DMSO, was equipped with a mechanical stirrer and was placed in an ambient temperature water bath. Aniline **S3** (15.1 g, 60.1 mmol) was dissolved in 100 mL of DMSO and was added to the NaSH suspension dropwise *via* cannula with positive N2 pressure over 1 h with mechanical stirring. The reaction mixture was stirred at ambient temperature under N2 for 16 h and was then placed in an ice water bath. The reaction was quenched with 50 mL of concentrated HCl and the resulting mixture was stirred for 45 min in the cold water bath. The reaction was neutralized to pH 8.0 with NaHCO3, first with 50 mL of a saturated aqueous solution, then with solid NaHCO3. The resulting mixture was diluted to 4 L with water, then was separated into two fractions of 2 L each. Each fraction was extracted with CH2Cl2 (4 × 1 L). The combined organic layer is concentrated to 2 L and was washed with saturated NaHCO3 (1 L), then with brine (1 L), then was dried over Na2SO4­ with stirring for 20 min. The solids were removed by filtration, and the volatile components were removed with a rotary evaporator with an ice water bath. The crude residue was purified *via* SiO2 flash chromatography with an eluent of 6:1 then 4:1 hexanes:MTBE to yield the product in two fractions. Other solvent systems (hexanes:DCM, hexanes:EtOAC, pentane:ether) were unsuccessful in separating the product from a very close impurity. Pure product was isolated as a yellow oil (8.49 g, 44%), and a second fraction of product (7.83 g) was isolated as an orange oil, which contains a small amount of a byproduct (<10%) and can be purified further. The NMR data of the purified product correlates with the published data .

**8-(Trifluoromethyl)-1,2,3,4,5-benzopentathiepin-6-amine hydrochloride (TC-2153).** Free aniline **S4** (23.3 g, 72.8 mmol) was dissolved in diethyl ether (220 mL, 0.33 M) in a 1 L glass beaker with a stir bar. Concentrated HCl (9.5 mL, 110 mmol, 1.5 equiv) was added dropwise and a yellow precipitate formed. The mixture was stirred at ambient temperature for 90 min, at white point the solid product was isolated *via* filtration. Drying over vacuum yields 18.28 g (70%) of the pure product as a light yellow solid. The filtrate was collected, concentrated, dissolved in 50 mL of ether, acidified with 1 mL of concentrated HCl and filtered to acquire a second crop of solid. The solid was washed with cold ether to remove any orange color, providing an additional crop of pure product (2.09 g, 8%), m.p. 142-147 °C (dec). 1H NMR (400 MHz, DMSO) δ 9.40-7.20 (br s, 3H), 7.17 (d, J = 2.0 Hz, 1H), 7.13 (d, J = 2.0 Hz, 1H). 19F NMR (376 MHz, DMSO) δ -62.43. 13C NMR (126 MHz, DMSO) δ 153.40, 146.21, 131.16 (d, J = 32.1 Hz), 124.75, 123.02 (q, J = 273.3 Hz), 117.29, 113.82 (d, J = 4.8 Hz). The NMR data of the purified product correlates with the published data .

**Results**

Eight compounds were selected for further characterization based on chemical structure and IC50 values, which ranged between 1 μM and 9.7 μM (Table S1). Preliminary studies of these molecules indicated potent inhibition of STEP activity in neuronal cultures and cortical tissue by one compound (Compound **1)** after intraperitoneal (i.p.) injections in WT mice.

However, as our supply of commercial inhibitor was depleted and was no longer available commercially, we synthesized Compound **1**. To our surprise, it showed greatly diminished activity. Several additional lead compounds were synthesized and all showed significantly lower STEP inhibition compared to the commercially purchased compounds(Figure S1).

**References**

1. Lipinski CA, Lombardo F, Dominy BW, Feeney PJ (2001) Experimental and computational approaches to estimate solubility and permeability in drug discovery and development settings. Adv Drug Deliv Rev 46: 3-26.

2. Kulikov AV, Tikhonova MA, Kulikova EA, Khomenko TM, Korchagina DV, et al. (2011) [Effect of new potential psychotropic drug, 8-(trifluoromethyl)-1,2,3,4,5-benzopentathiepin-6-amine hydrochloride, on the expression of serotonin-related genes in mouse brain]. Mol Biol (Mosk) 45: 282-288.

3. Gao Y, Ren Q, Wu H, Li M, Wang J (2010) Enantioselective heterocyclic synthesis of spiro chromanone-thiochroman complexes catalyzed by a bifunctional indane catalyst. Chemical communications (Cambridge, England) 46: 9232-9234.

4. Mutlib A, Espina R, Atherton J, Wang J, Talaat R, et al. (2012) Alternate strategies to obtain mass balance without the use of radiolabeled compounds: application of quantitative fluorine (19F) nuclear magnetic resonance (NMR) spectroscopy in metabolism studies. Chemical research in toxicology 25: 572-583.
